# Global Epidemiology of Dengue Outbreaks in 1990–2015: A Systematic Review and Meta-Analysis

**DOI:** 10.3389/fcimb.2017.00317

**Published:** 2017-07-12

**Authors:** Congcong Guo, Zixing Zhou, Zihao Wen, Yumei Liu, Chengli Zeng, Di Xiao, Meiling Ou, Yajing Han, Shiqi Huang, Dandan Liu, Xiaohong Ye, Xiaoqian Zou, Jing Wu, Huanyu Wang, Eddy Y. Zeng, Chunxia Jing, Guang Yang

**Affiliations:** ^1^Department of Epidemiology, School of Medicine, Jinan University Guangzhou, China; ^2^Department of Parasitology, School of Medicine, Jinan University Guangzhou, China; ^3^Department of Viral Encephalitis, Chinese Center for Disease Control and Prevention, Institute for Viral Disease Control and Prevention, National Institute for Viral Disease Control and Prevention Beijing, China; ^4^State Key Laboratory for Infectious Disease Prevention and Control, Chinese Center for Disease Control and Prevention Beijing, China; ^5^Guangzhou Key Laboratory of Environmental Exposure and Health, Guangdong Key Laboratory of Environmental Pollution and Health, School of Environment, Jinan University Guangzhou, China

**Keywords:** dengue, outbreak, epidemiology, clinical characteristic, risk factor, serotype, systematic review, meat-analysis

## Abstract

Dengue is an arthropod-borne infectious disease caused by dengue virus (DENV) infection and transmitted by *Aedes* mosquitoes. Approximately 50–100 million people are infected with DENV each year, resulting in a high economic burden on both governments and individuals. Here, we conducted a systematic review and meta-analysis to summarize information regarding the epidemiology, clinical characteristics, and serotype distribution and risk factors for global dengue outbreaks occurring from 1990 to 2015. We searched the PubMed, Embase and Web of Science databases through December 2016 using the term “dengue outbreak.” In total, 3,853 studies were identified, of which 243 studies describing 262 dengue outbreaks met our inclusion criteria. The majority of outbreak-associated dengue cases were reported in the Western Pacific Region, particularly after the year 2010; these cases were primarily identified in China, Singapore and Malaysia. The pooled mean age of dengue-infected individuals was 30.1 years; of the included patients, 54.5% were male, 23.2% had DHF, 62.0% had secondary infections, and 1.3% died. The mean age of dengue patients reported after 2010 was older than that of patients reported before 2010 (34.0 vs. 27.2 years); however, the proportions of patients who had DHF, had secondary infections and died significantly decreased after 2010. Fever, malaise, headache, and asthenia were the most frequently reported clinical symptoms and signs among dengue patients. In addition, among the identified clinical symptoms and signs, positive tourniquet test (*OR* = 4.86), ascites (*OR* = 13.91) and shock (*OR* = 308.09) were identified as the best predictors of dengue infection, DHF and mortality, respectively (both *P* < 0.05). The main risk factors for dengue infection, DHF and mortality were living with uncovered water container (*OR* = 1.65), suffering from hypotension (*OR* = 6.18) and suffering from diabetes mellitus (*OR* = 2.53), respectively (all *P* < 0.05). The serotype distribution varied with time and across WHO regions. Overall, co-infections were reported in 47.7% of the evaluated outbreaks, and the highest pooled mortality rate (2.0%) was identified in DENV-2 dominated outbreaks. Our study emphasizes the necessity of implementing programs focused on targeted prevention, early identification, and effective treatment.

## Introduction

Only 9 countries had experienced severe dengue epidemics before 1970; however, at present, dengue fever has affected more than 100 countries in tropical and subtropical regions (Organization, 2016). It was estimated by WHO that 50–100 million dengue infections occur annually, with a 30-fold increase in global incidence observed over the past 50 years (WHO, 2012). Today, dengue virus (DENV) poses a major threat to global public health, and approximately two-fifths of the world's population is at risk of dengue infection (Lancet, 2013; Screaton et al., 2015).

Since the first dengue outbreak was reported in 1779 in Jakarta, Indonesia (Wu et al., 2011), this disease has become a public health threat that is associated with remarkable morbidity and mortality. The case fatality rate in untreated dengue patients has been reported to reach 20%, but this rate can be reduced to less than 1% under expert clinical management and with careful fluid replacement (Simmons et al., 2012). The number of dengue outbreaks caused by the four dengue virus serotypes (DENV-1 to DENV-4) has increased since 1980, mainly affecting Asia, South America and the Caribbean (Jansen and Beebe, 2010; Amarasinghe et al., 2011). The various serotypes of the DENV are transmitted predominantly by the mosquito vector, *Aedes aegypti* (Guzman and Harris, 2015); however, DENV can also be transmitted by other species of the genus *Aedes*, including *Aedes albopictus* (Barcelos, 2014). While most dengue patients recover after experiencing self-limiting illness, a small proportion progress to dengue hemorrhagic fever (DHF) (WHO/TDR, 2009). DHF may be classified into four severity grades, with grades III and IV being defined as dengue shock syndrome (DSS) (WHO/TDR, 2009). Although the characteristics of dengue infection have been well described, previous studies have mainly focused on the descriptions of single outbreaks, and few of studies have concentrated on systematically summarizing the epidemiological characteristics of dengue outbreaks worldwide. In this situation, it may difficult to keep abreast of the trends of global dengue outbreaks.

At present, little attention has been specifically paid to summarizing global dengue outbreaks. We performed a robust systematic analysis of all available data to gain a better understanding of the global epidemiology, clinical manifestations, and serotype distribution and risk factors for dengue outbreaks.

## Materials and methods

### Searching strategy and selection criteria

**Figure 1:**
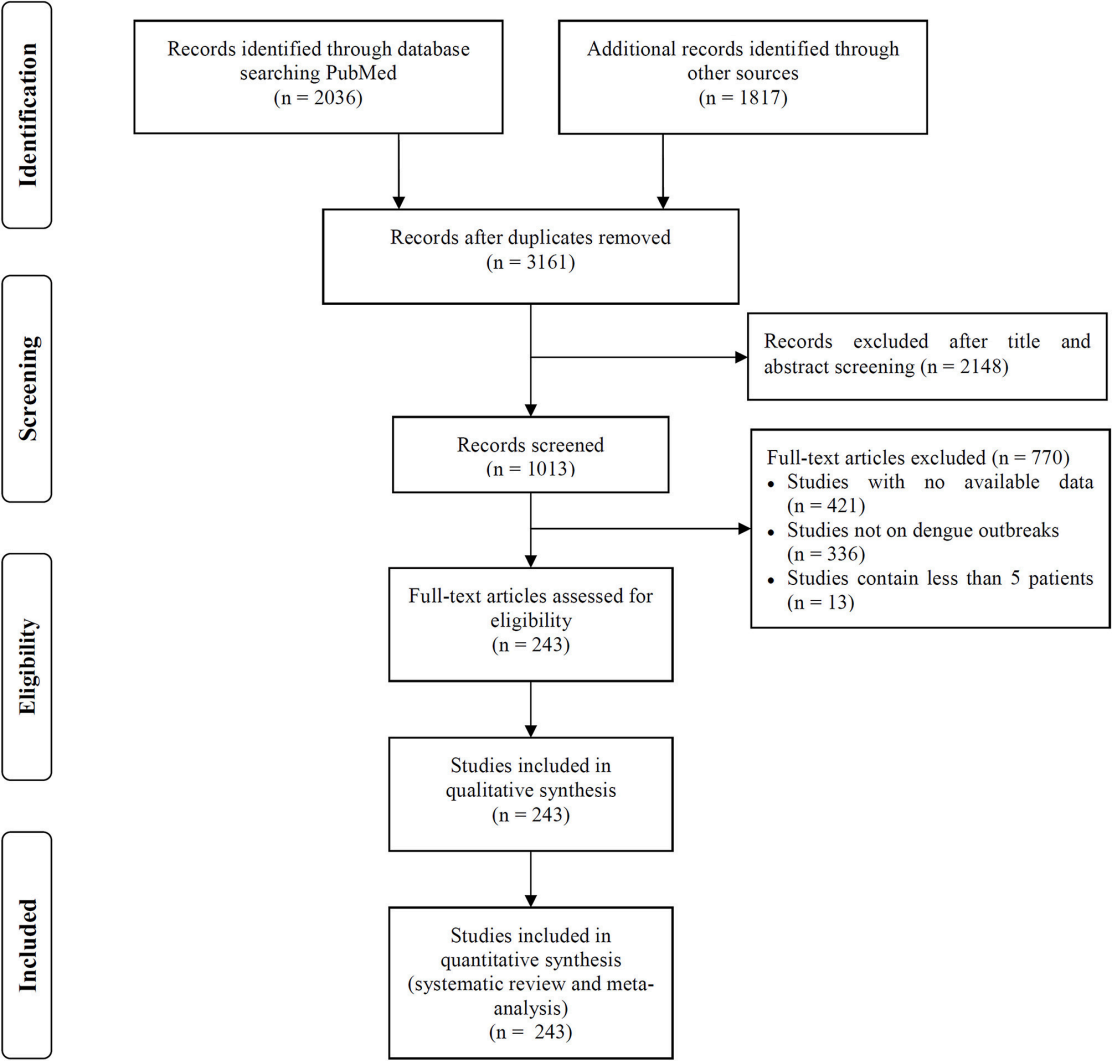
Flowchart of literature selection process in this study.

We searched the PubMed, Embase and Web of Science databases to identify articles describing dengue outbreaks that occurred between Jan 1, 1990 to Dec 1, 2016 (Figure 1). The following search terms were used as a text word in each database: PubMed, “dengue outbreak” in all fields; limited to human studies; Embase, “dengue outbreak” in all fields; limited to human studies; and Web of Science, “dengue outbreak” in the topic field with the exclusion of veterinary studies. Two independent reviewers (C.C.G and Z.X.Z) screened the titles and abstracts of all related manuscripts, searched reference lists of the identified studies and obtained full texts for potentially relevant articles. Studies without any dengue outbreak data available, that described outbreaks including less than 5 dengue patients or that did not focus on a specific dengue outbreak were excluded. When different articles described the same outbreak but provided different data, all studies were included.

### Data extraction

The following data were independently extracted from each included study by two investigators (C.C.G and Z.X.Z): author, year of publication, year of outbreak, WHO region, country, city, mosquito species, patient characteristics (including demographic characteristics, risk factors, clinical symptoms and number of secondary cases), dengue serotype, number of patients (including DF, DHF, and DSS patients), and number of deaths. Any disagreements were adjudicated by a senior investigator (C.X.J).

### Analysis

We described the epidemiology, clinical manifestations, risk factors and serotype distribution using the number of studies and cases, which are expressed as proportions with 95% CIs for categorical variables (sex, dengue case classification, primary or secondary infection, signs, symptoms and mortality) and means and corresponding 95% CIs for continuous variables (age). We compared the epidemiologic characteristics of dengue outbreaks occurring before 2010 and after 2010 (including 2010) using two-proportion z-tests for categorical variables and Student's *t*-tests for continuous variables. Meta-analyses were performed using Comprehensive Meta-Analysis version 2.2.064 software (Biostat Inc., NJ, USA). We assessed the level of heterogeneity across studies using *P*_*h*_ and *I*^2^ (Higgins and Thompson, 2002). If a *P*_*h*_-value was greater than 0.10, a fixed-effects model was used; otherwise, a random-effects model was selected (Handoll, 2006). The meta-analysis corresponded with recommendations from the Preferred Reporting Items for Systematic Reviews and Meta-Analyses (PRISMA) statement (Supplementary File S2). Medians (ranges) were converted to means (SDs) using previously proposed formulas (Hozo et al., 2005). Distribution maps for global dengue outbreaks and serotypes were generated in R software, version 3.3.1 (R Core Team, Vienna, Austria), using ggplot2, maps, mapproj and sp packages. The correlations between the rates of mortality, DHF, and secondary infections were assessed by generating Spearman correlation coefficients.

## Results

### Systematic review

**Figure 2:**
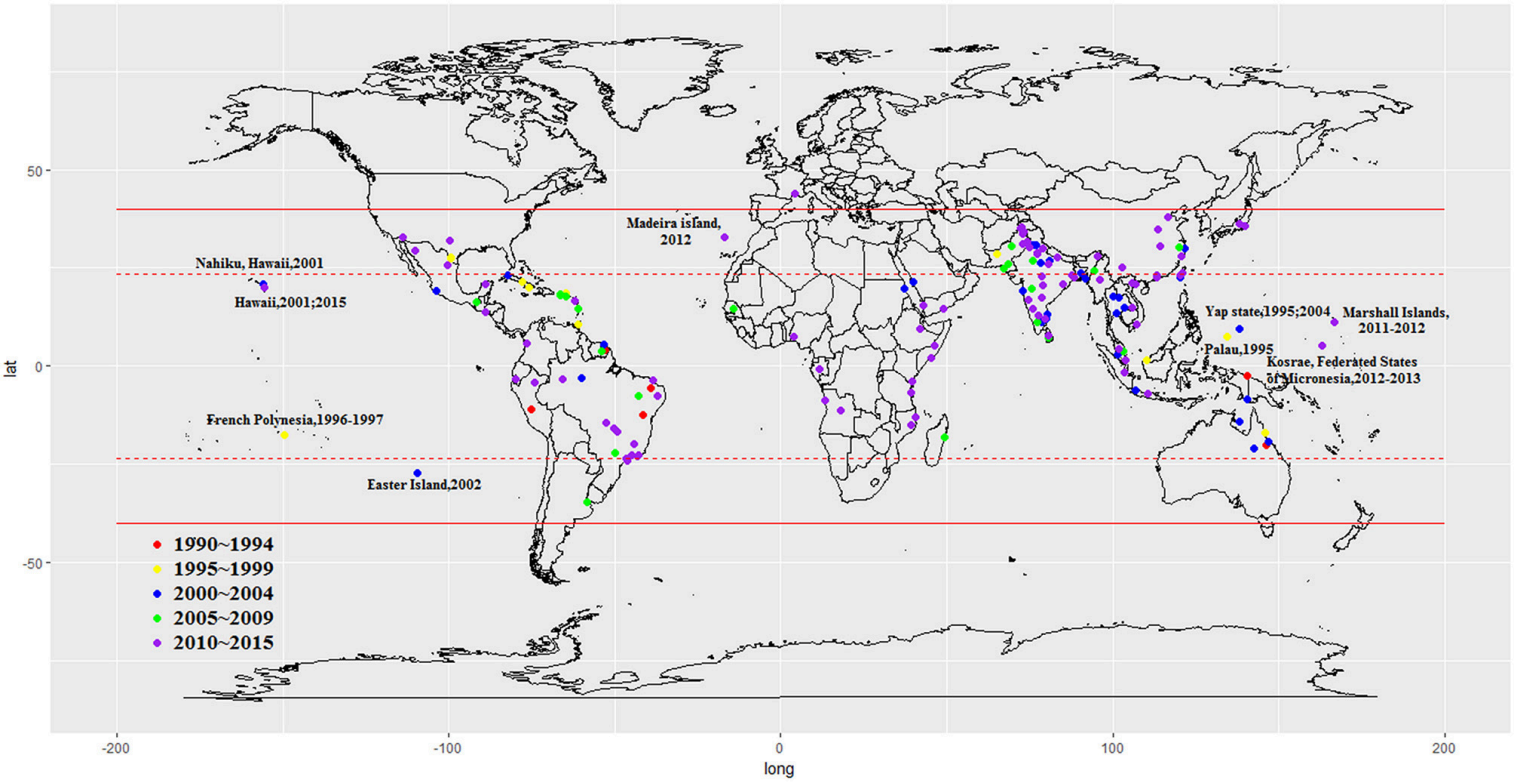
Global dengue outbreaks distribution from 1990–2015.

We included 243 articles describing 262 dengue outbreaks that occurred between 1990 and 2015 (Figure 1, Supplementary Table 1, Supplementary File S1). One hundred and twelve outbreaks described in 104 articles occurred after 2010. Among countries worldwide, the highest numbers of outbreak (58/262) are observed in India, followed by China (38/262) and Brazil (24/262) from 1990 to 2015. All the outbreaks occurred in tropical (77/262) and subtropical (174/262) regions, except for one outbreak, which occurred in Nîmes, 2015 (Succo et al., 2016), and was considered the first considerable dengue outbreak in mainland France (Figure 2). Among the six WHO regions, the largest number of outbreaks occurred in the Southeast Asia region (82/262), followed by the Western Pacific region (72/262) and the American region (65/262), accounting for more than 83.6% of outbreaks overall. The European region (6/262) was least affected by dengue outbreaks, with only 4 outbreaks reported in France (three in overseas departments and regions of France and one in mainland France) and 2 outbreaks reported in Portugal. However, the Western Pacific Region had most dengue outbreaks reported after 2010 (Western Pacific: 33/112 > Southeast Asia: 27/112 > Americas: 24/112 > Eastern Mediterranean: 13/112 > Africa: 11/112 > Europe: 4/112).

### Epidemiology

By the end of 2016, a total of 291,964 outbreak-associated dengue cases had been reported in the literature, mainly from China (27.9%), Singapore (27.0%) and Malaysia (15.1%). The majority (72.4%) of dengue patients were reported in the Western Pacific region, followed by the American region (19.4%), Southeast Asia Region (4.8%), Eastern Mediterranean region (1.5%), European region (1.5%) and African region (0.3%). Outbreaks occurring before 2010 (Americas > Western Pacific > South East Asia > Eastern Mediterranean > Europe > Africa) accounted for 28.6% of the total number of cases, the majority of which were reported in Cuba (28.1%), Singapore (19.3%) and Puerto Rico (13.4%), while patients in outbreaks after 2010 (Western Pacific > Americas > South East Asia > Europe > Eastern Mediterranean > Africa) were mainly from China (36.8%), Singapore (30.1%), and Malaysia (20.9%). 50.0% outbreaks occurred in urban areas (21/42), 28.6% in rural areas (12/42) and 21.4% in both urban and rural areas (9/42). It is important to note that nearly all rural outbreaks occurred after 2000 (except one in Malaysia, 1999; Cheah et al., 2006). It is consistent with the view that dengue once confined to urban areas has penetrated into the rural setup (Ishak et al., 1997). The improved road systems, better socio-economic situations and established agricultural settlements in rural areas may increase the Ae. albopictus population, and thus spread of rural dengue fever among the rural communities (Chang et al., 1997).

**Figure 3:**
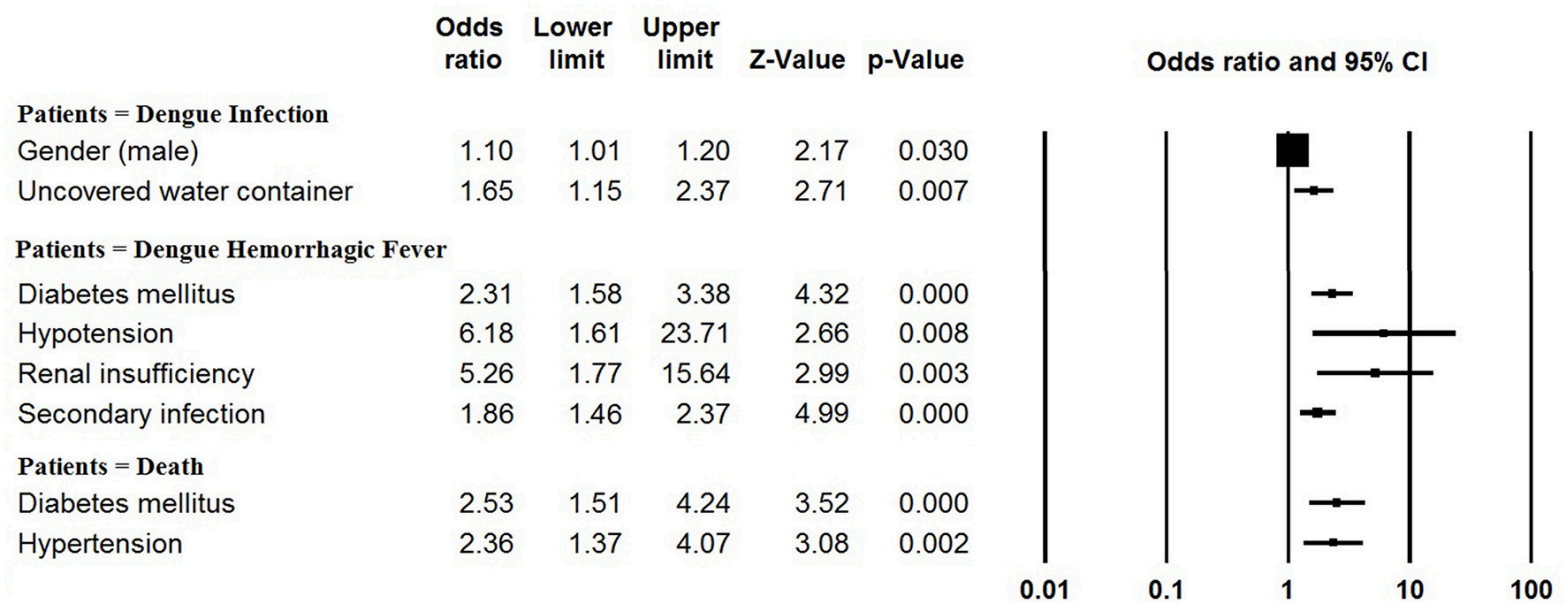
Meta-analysis: forest plot of the associations between risk factors and dengue infection, DHF and mortality in dengue outbreaks.

The pooled mean age of the patients was 30.1 years, and 54.5% were male (Table 1, Supplementary Table 2). The results of the meta-analyses indicated there to be significant associations between dengue infection and two variables: male gender (OR: 1.10, 95% CI: 1.01–1.20) and living with uncovered water container (OR: 1.65, 95% CI: 1.15–2.37) (Figure 3, Supplementary Table 3). The pooled rate of DHF was 23.2%; secondary infection (OR: 1.86, 95% CI: 1.46–2.37), diabetes mellitus (OR: 2.31, 95% CI: 1.58–3.38), hypotension (OR: 6.18, 95% CI: 1.61–23.71) and renal insufficiency (OR: 5.26, 95% CI: 1.77–15.64) were at increased odds of DHF (Figure 3, Supplementary Table 4). The rate of secondary infections among dengue patients was 62.0% in the meta-analysis of the 35 studies reporting these data; patients with a platelet count < 100 × 10^9^/L had increased odds of secondary infection (OR: 4.11, 95% CI: 1.64–10.31) (Supplementary Table 5). In addition, the pooled rate of mortality derived from the 72 studies reporting this outcome was 1.3%; patients with underlying diseases, such as diabetes mellitus (OR: 2.53, 95% CI: 1.51–4.24) and hypertension (OR: 2.36, 95% CI: 1.37–4.07) were at increased odds of mortality (Figure 3, Supplementary Table 6).

**Table 1:** Epidemiologic factors of dengue patients in global outbreaks.

**Variables**	**No. studies meta-analyzed**	Meta-analysis, pooled data (95% CI)^*^	***P* value**
Mean age	96	30.1 (27.7–32.5)	0.006
Before 2010	55	27.2 (24.5–30.0)	
After 2010	41	34.0 (30.1–38.0)	
Male (%)	146	54.5 (53.2–55.7)	0.194
Before 2010	83	54.7 (52.6–56.7)	
After 2010	63	54.3 (52.5–56.0)	
DHF (%)	107	23.2 (18.3–29.0)	<0.001
Before 2010^a^	85	18.8 (14.3–24.3)	
After 2010^a^	22	15.7 (7.6–29.6)	
Secondary infection (%)	35	62.0 (53.5–69.8)	<0.001
Before 2010	20	66.7 (55.1–76.6)	
After 2010	15	55.9 (43.8–67.3)	
Fatal cases (%)	72	1.3 (0.9–2.0)	<0.001
Before 2010	46	1.9 (1.2–2.9)	
After 2010	26	0.7 (0.3–1.8)	

* *Random-effects model unless otherwise specified*.

a *Removed articles contained all DHF patients; P-value, two-proportion z-test for categorical factors and Student's t-test for continuous factors*.

Patient age distributions, DHF rates, secondary infection rates and mortality rates differed significantly between outbreaks occurring before the year 2010 and occurring after 2010 (Table 1). Dengue patients identified in association with outbreaks occurring after 2010 were significantly older than those identified in association with outbreaks occurring before 2010 (mean, 95% CI: 34.0, 30.1–38.0 vs. 27.2, 24.5–30.0; *P* = 0.006). The rates of DHF, secondary infection and mortality significantly decreased after 2010 (all *P* < 0.001). No significant gender difference was identified between these two time periods (*P* = 0.194). The rate of DHF and rate of mortality observed in the included dengue outbreaks were significantly correlated, with an overall Spearman correlation coefficient of 0.64 (*P* < 0.001). However, no significant correlation was observed between the rate of secondary infection and the rate of mortality (*P* = 0.223).

### Clinical symptoms and signs

Fever was the most commonly observed clinical symptom or sign (pooled proportion: 98.1, 95% CI: 97.2–98.7%), followed by malaise (76.0, 95% CI: 64.1–84.9%), headache (75.7, 95% CI: 69.5–81.0%) and asthenia (74.3, 95% CI: 45.8–90.8%), which are listed in Table 2 (Supplementary Table 7). Hemorrhagic manifestations were observed in 25.8% (95% CI: 21.0–31.1%) of the patients, of which petechiae (22.3, 95% CI: 16.5–29.3%) was the most common.

**Table 2:** Clinical symptoms and signs of patients in dengue outbreaks.

**Variables**	**No. studies meta-analyzed**	Meta-analysis, pooled data (95% CI)^*^
**CONSTITUTIONAL**		
Fever	88	98.1 (97.2–98.7)
Chills	14	65.3 (58.3–71.6)
Myalgia	65	64.2 (58.1–69.8)
Arthralgia	53	53.6 (46.0–61.0)
Lethargy	5	67.1 (32.6–89.6)
Malaise	9	76.0 (64.1–84.9)
Asthenia	6	74.3 (45.8–90.8)
Body-ache	13	67.2 (55.2–77.3)
Back pain	9	57.3 (32.2–79.1)
Sore throat	12	19.7 (13.4–28.1)
Eye pain	6	27.8 (13.7–48.1)
Retro-orbital pain	38	35.1 (27.0–44.2)
Lymphadenopathy	13	9.2 (4.4–18.2)
**GASTROINTESTINAL**		
Vomiting	36	39.8 (35.0–44.9)
Nausea	22	42.0 (34.0–50.4)
Diarrhea	36	20.7 (17.3–24.7)
Anorexia	17	47.8 (34.9–61.0)
Ascites	25	10.2 (5.3–18.8)
Icterus/Jaundice	13	2.8 (1.5–5.2)
Abdominal pain	61	32.4 (27.9–37.2)
Hepatomegaly	41	18.9 (12.7–27.1)
Splenomegaly	20	7.7 (5.2–11.3)
Hepatosplenomegaly	5	17.5 (8.3–33.3)
**MUCOCUTANEOUS**		
Rash	83	29.6 (26.1–33.3)
Pruritus	5	24.1 (19.8–29.0)
Exanthema	5	33.7 (11.2–67.1)
Itching eruption	6	24.0 (18.7–30.2)
**CARDIORESPIRATORY**		
Cough	29	22.9 (17.8–28.8)
Pleural effusion	23	8.3 (4.5–14.9)
Myocarditis	5	5.7 (1.2–22.5)
Hypotension	14	12.5 (7.7–19.7)
Respiratory disorders	13	8.7 (5.3–13.9)
**NEUROLOGICAL**		
Headache	83	75.7 (69.5–81.0)
Dizziness	9	22.8 (11.7–39.7)
Seizure	7	2.7 (1.8–3.9)^**^
Shock	12	9.5 (4.2–20.0)
Convulsion	5	6.1 (2.9–12.5)
Encephalopathy	8	5.0 (1.9–12.4)
**HEMORRHAGIC MANIFESTATIONS**		
Gingivorrhagia	16	9.7 (6.0–15.2)
Epistaxis	25	11.8 (7.6–17.9)
Hematuria	16	5.0 (3.0–8.1)
Melena	13	16.9 (8.1–31.8)
Petechiae	30	22.3 (16.5–29.3)
Hematemesis	18	13.4 (8.0–21.6)
Bleeding/Hemorrhagic manifestations	58	25.8 (21.0–31.1)

* *Random-effects model unless otherwise specified*.

** *Fixed-effects model*.

**Figure 4:**
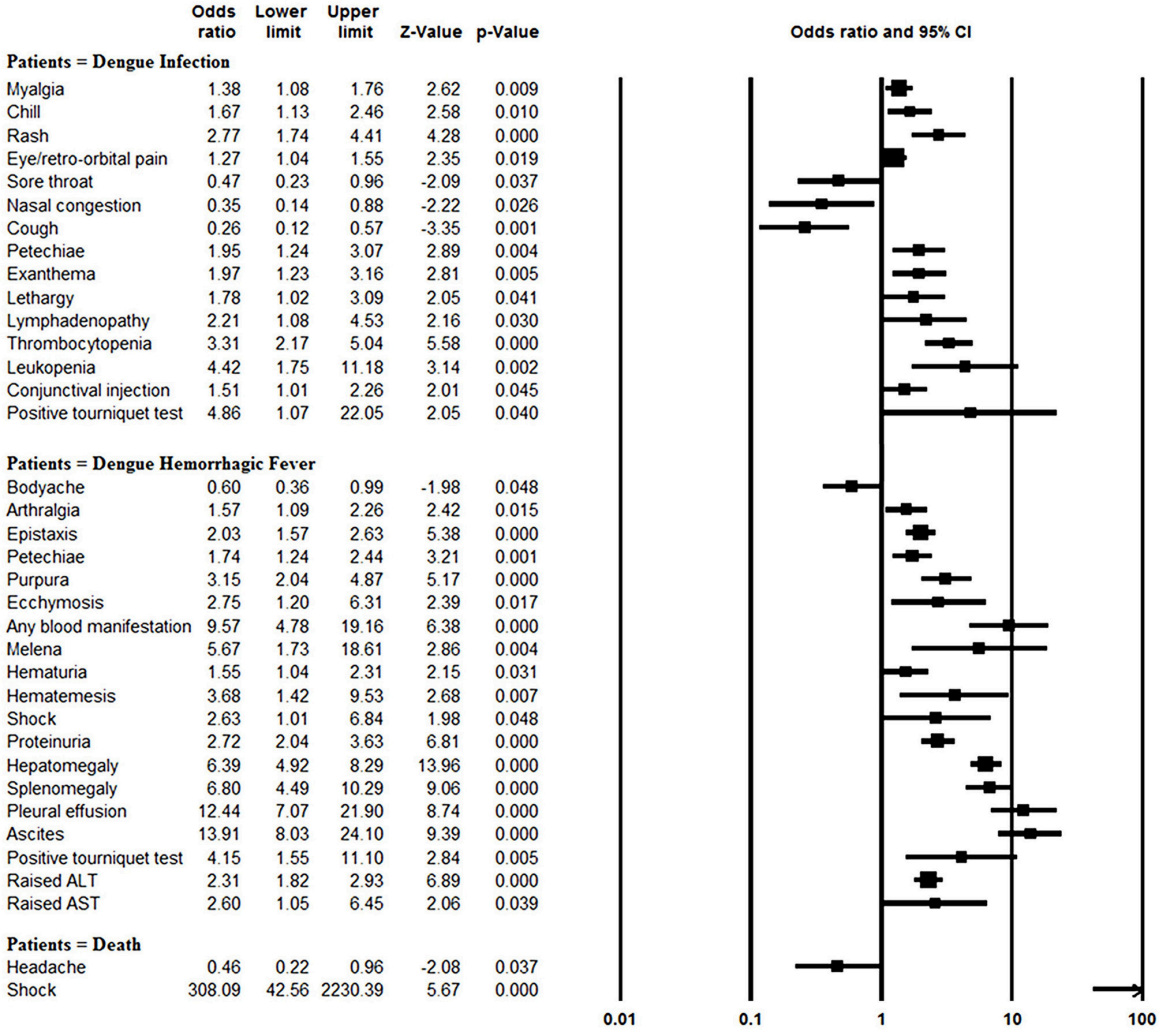
Meta-analysis: forest plot of the associations between symptoms and signs and dengue infection, DHF and mortality in dengue outbreaks.

The rates of myalgia, chill, rash, eye/retro-orbital pain, petechiae, exanthema, lethargy, lymphadenopathy, thrombocytopenia, leukopenia, conjunctival injection, and positive tourniquet test results were significantly greater in the dengue-infected group than the laboratory negative group (all *P* < 0.05). On the other hand, since most of the laboratory negative patients were infected with influenza, those patients more frequently suffered from sore throat, nasal congestion and cough than did dengue-infected patients (all *P* < 0.05) (Figure 4, Supplementary Table 3).

Blood manifestations (OR: 9.57, 95% CI: 4.78–19.15), pleural effusion (OR: 12.44, 95% CI: 7.07–21.91), ascites (OR: 13.91, 95% CI: 8.03–24.11) and morality (OR: 11.46, 95% CI: 4.16–31.56) were more common in DHF patients than DF patients, and other symptom and sign comparisons are shown in Figure 4, Supplementary Table 4. However, body-ache (OR: 0.60, 95% CI: 0.36–0.99) was identified significantly less frequently in DHF patients than DF patients.

Patients with shock symptom were at significantly increased odds of mortality (OR: 308.09, 95% CI: 42.56–2230.53). In contrast, headaches were negatively associated with mortality (OR: 0.46, 95% CI: 0.22–0.95) (Figure 4, Supplementary Table 6).

### Serotype

Studies of 174 outbreaks that occurred between 1990 and 2015 reported dengue serotype data. The highest number of monoinfection outbreaks were caused by DENV-2 (36, 20.7%), followed by DENV-1 (29, 16.7%), DENV-3 (19, 10.9%) and DENV-4 (7, 4.0%). Coinfection with more than one DENV serotype was reported in 47.7% of the outbreaks; outbreaks involving all four serotypes were the most common (25, 14.4%), followed by coinfection with DENV-1 and DENV-2 (16, 9.2%) and coinfection DENV-1, DENV-2 and DENV-3 (12, 6.9%).

**Figure 5:**
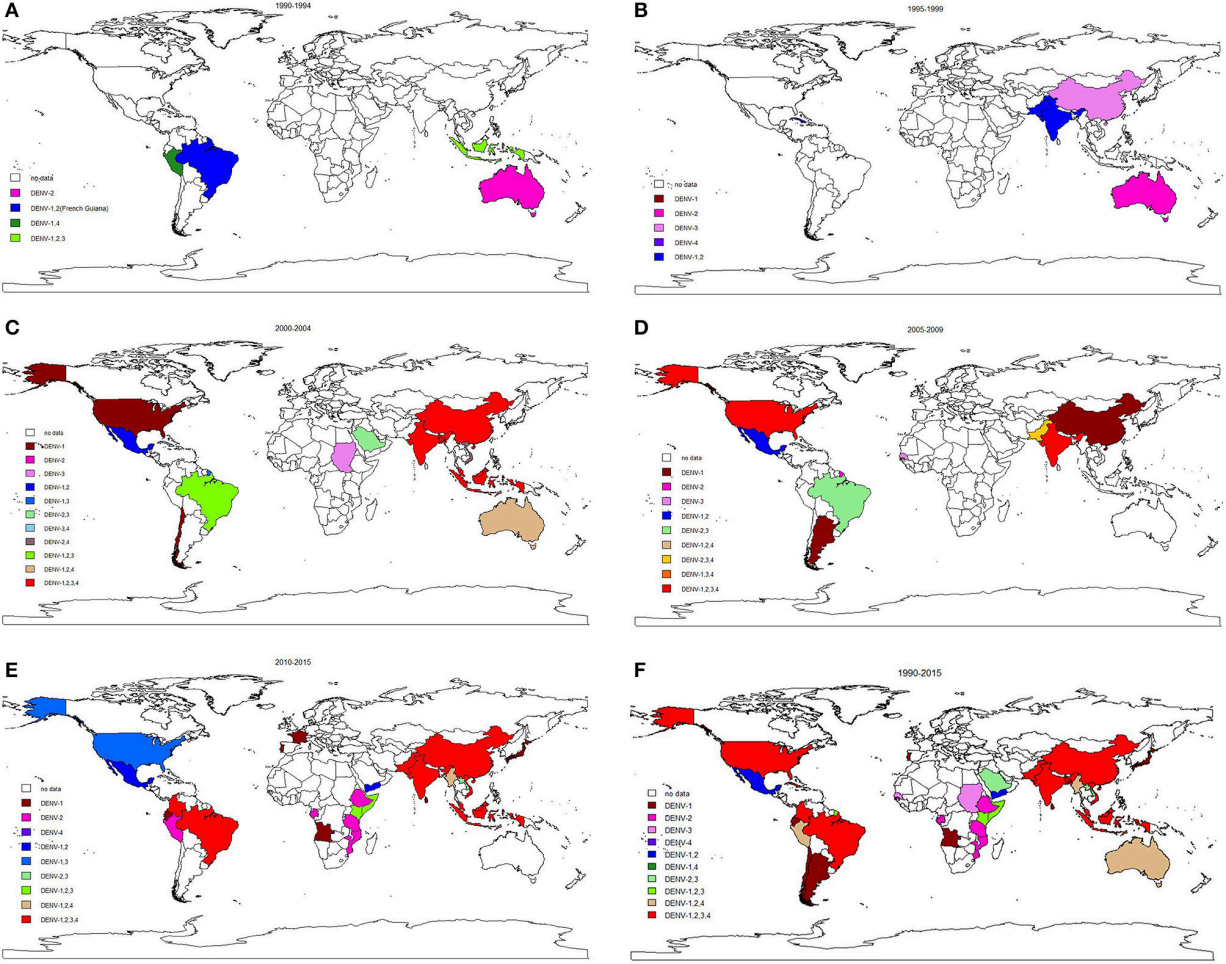
Global serotypes distribution in dengue outbreaks from 1990–2015.

The longitudinal trends in serotype distribution are shown in Figure 5. Between 1990 and 1994, DENV-2 was most frequently identified serotype in the 6 reported outbreaks (5/6, 83.3%). During 1995–1999 and 2000–2004, DENV-2 monoinfection was the predominant serotype observed in dengue outbreaks (11/20, 55.0% and 10/45, 22.2%). Coinfection dominated subsequent (2005–2009) outbreaks (12/23, 52.2%), especially co-infection with all four serotypes (4/23, 17.4%). After 2010, DENV-1 dominated the monoinfection outbreaks (17/34, 50.0%) and co-infection with all four serotypes continued to dominate the coinfection outbreaks (17/46, 37.0%). Different serotypes were predominant in different WHO regions after 2010: DENV-1 and DENV-2 were most common in the African region and American region; DENV-1 was most common in the European region; coinfection with all four serotypes was most common in the Southeast Asia region; DENV-2 and DENV-3 were most frequently observed in the Eastern Mediterranean region, and DENV-1 was predominant in the Western Pacific region.

The highest pooled mortality rate was identified in DENV-2 dominated outbreaks at 2.0% (95% CI: 0.9–4.2%), followed by DENV-3 (1.6, 95% CI: 0.4–6.8%), DENV-4 (0.7, 95% CI: 0.0–42.3%) and DENV-1 (0.3, 95% CI: 0.1–0.9%).

## Discussion

Our study presents the epidemiology, clinical characteristics, and serotype distribution and risk factors for global dengue outbreaks in humans that occurred between 1990 and 2015. The majority of the 262 outbreaks occurred in developing countries, including India, China and Brazil. These high numbers might be attributed to the limited laboratory facilities and inadequate control measures in these countries (Cattand et al., 2006). The relatively low socioeconomic status of and high population density and ideal environment for the maintenance of mosquitoes in developing countries may also facilitate the transmission of DENV (Figueiredo, 2007; Saswat et al., 2015). After 2010, the majority of dengue outbreaks and dengue patients were identified in the Western Pacific region, which may be mainly attributed to cases in China, Singapore and Malaysia. In contrast, only a few dengue outbreaks were reported in the Europe region, and these outbreaks involved in only two countries (France and Portugal). The disappearance of *A. aegypti* from the European Basin between the 1950s and 2005 might be one reason for its low-prevalence of dengue outbreaks (Succo et al., 2016). It is important to note that many dengue outbreaks were influenced by the dengue epidemics in nearby/neighboring countries. Wang et al. reported that the dengue virus in the Xishuangbanna and Dehong, China outbreak in 2013 was imported from Southeast Asia countries (Wang et al., 2016). Owing to the more frequent interactions between the populations of Southeast Asian countries and China, imported dengue epidemics have been documented in southern China. Additionally, the first major outbreak of dengue in Europe was reported to be most likely imported from Venezuela via travelers to Madeira (Wilder-Smith et al., 2014). Valid laboratory-based disease surveillance system and integrated vector management are required in the border regions of dengue epidemic areas so that future disease outbreaks and spread to other regions can be prevented (Guo et al., 2015).

Ongoing outbreaks likely reflect deficiencies in vector control and prevention. Originating in Africa and spreading to tropical countries in the seventeenth and eighteenth centuries, *A. aegypti* has been considered the primary dengue vector due to its enhanced viral duplication capability, thus increasing the probability of viral transmission (Gubler, 2011; Chepkorir et al., 2014; Whitehorn et al., 2015). Sixty of the articles included in our meta-analysis mentioned mosquitoes. Consistent with prior knowledge, the most prevalent mosquito was *A. aegypti* (76.7%), followed by *A. albopictus* (43.3%). In addition, the pooled mortality rate in outbreaks associated with *A. aegypti* mosquitoes (0.7, 95% CI: 0.3–1.7%) was higher than the pooled mortality rate in outbreaks associated with *A. albopictus* mosquitoes (0.4, 95% CI: 0.1–2.5%). Dengue outbreaks caused by *A. albopictus* tend to be mild and short (Issack et al., 2010). Wang T. et al. (2015), Wang S. F. et al. (2015), Kim Lien et al. (2015), Kunwar and Prakash (2015), Barde et al. (2015) had reported that rainfall with the hot weather contributed to the dramatically increase of mosquitos. Rainfall provides breeding sites and stimulates egg hatching for dengue transmission vectors and temperature affects the vector's survival and their rate of development and reproduction (Johansson et al., 2009). Chepkorir et al. (2014) and Watts et al. (1987) have demonstrated a significantly higher infection rate of dengue virus at high temperatures for Ae. aegypti mosquitoes, suggesting a potentially significant role of temperature in the dynamics of dengue transmission. It may be due to that the high temperature increases virus reproduction to high titers and reduces the extrinsic incubation period for the dengue virus to be established within the vector, thus leads to the upsurge of dengue outbreaks (Lambrechts et al., 2011). Besides the traditional methods to control vectors, a potential method has been developed during the last decade; scientists released *A. aegypti* or *A. albopictus* mosquitoes infected with *Wolbachia*, which might hinder the insects' ability to transmit the dengue virus and cannot infect humans (Hoffmann et al., 2011; Callaway, 2016).

The results of our study suggested that DENV-2 was the predominant serotype in dengue outbreaks that occurred before 2000, but DENV-3 was the predominant serotype between 2000 and 2009. After 2010, DENV-1 dominated global dengue outbreaks, and DENV-4 was the least frequently identified serotype. We would like to note that at least two serotypes were identified in nearly half the outbreaks, as previous exposure to a single virus could not provide immunity against potential infections with other serotypes (Rahim and Sikder, 2005). Cross-reactive and non-neutralizing antibodies from the primary infection can also bind to the new DENV serotype and facilitate virus entry into susceptible cells. It was a phenomenon known as antibody-dependent enhancement of infection (ADE), considered as the most rational explanation for severe dengue (Simmons et al., 2007; Wang Y. et al., 2015). This can also explain the significantly association between secondary infection and DHF in our study (OR: 1.86, 95% CI: 1.46–2.37). Additionally, we found that the pooled mortality rate was highest in DENV-2 dominated outbreaks (2.0%), with significantly higher mortality rates identified in association with this than other serotypes. Although vaccines or therapeutics for dengue are not available, Liu *et al*. previously identified multiple mosquito galactose specific C-type lectins (mosGCTLs) that facilitated dengue infection; these mosGCTLs were induced in the tissues of mosquitoes with DENV-2 and directly interacted with the DENV-2 surface envelope (E) protein and virions *in vitro* and *in vivo*. Membrane blood feeding of antisera against mosGCTLs has been found to efficiently reduce DENV-2 infections among mosquitoes, suggesting that immunization against mosGCTLs may serve as a feasible approach for preventing dengue infection (Liu et al., 2014). Additionally, to reduce the rate of mortality in dengue patients, the application of early clinical and laboratory diagnosis, intravenous rehydration, staff training and hospital reorganization should be ensured during outbreaks (Lo et al., 2007; WHO, 2012).

Dengue infections were characterized by fever (98.1%), malaise (76.0%), headache (75.7%), and asthenia (74.3%); however, in some cases, bleeding (25.8%), plasma leakage (8.3%) and organ impairment (e.g., hepatosplenomegaly, 17.5%) were observed. Although dengue was mostly mild and self-limiting in this study, when cases were misdiagnosed by untrained physicians, there may be sufficient time for virus transmission and outbreak development (Huang Xue et al., 2014). In addition to the common symptoms and signs of dengue, manifestations including bleeding (OR: 9.57), pleural effusion (OR: 12.44), and ascites (OR: 13.91) were frequently identified in DHF patients. Thus, physicians should monitor patients for hemorrhage manifestations and capillary leakage, which might indicate the onset of DHF or DSS (Trofa et al., 1997). The results of our study showed that the rates of DHF, secondary infection and mortality in dengue outbreaks were significantly lower in outbreaks occurring after 2010 than in outbreaks before 2010, which suggests that the severity of global dengue infection has somewhat decreased after 2010. Through the use of timely, appropriate clinical management, global outbreak surveillance and sustainable vector control, a great deal of effort has been devoted to achieving the goal set by the World Health Organization in 2012; however, these efforts still face a challenging situation (WHO, 2012).

We believe it is important to note that the age of dengue patients after 2010 was significantly older than that of dengue patients before 2010 (mean pooled age: 34.0 vs. 27.2). Possible reasons for this finding may be that younger people now spend more daytime in enclosed air-conditioned environments and are, therefore, less likely to be exposed to mosquitoes (Lin et al., 2012) and that older people with chronic diseases now visit the doctor more often, causing existing dengue infection to be more likely to be detected (Lee et al., 2006).

There are still some limitations to our study. First, since our study and analysis were based on published articles, some dengue outbreaks may not have been recorded; thus, the result of our study should be interpreted with caution. Second, dengue is a self-limiting disease and most infections are asymptomatic (Bordignon et al., 2008); therefore, a portion of dengue patients might go unidentified during dengue outbreaks. The actual numbers of cases associated with these outbreaks were likely considerably higher than recorded. Regardless of these limitations, we believe that our systematic review and meta-analysis provide useful information regarding the global epidemiology of dengue outbreaks. Our study indicated the countries and WHO regions most seriously affected by dengue outbreaks; the risk factors for and clinical characteristics of dengue infection, DHF and mortality; and the global dengue serotype distribution, all of which could be extremely valuable when targeting prevention and early identification efforts in dengue outbreaks worldwide.

## Author contributions

GY and CJ contributed to the design of the study. CG and ZZ were involved in data acquisition. The data was then analyzed and interpreted by all authors. CG, ZZ, ZW, and YL wrote the first manuscript. GY and CJ critically revised the manuscript for important intellectual content. All authors approved the final version to be submitted.

### Conflict of interest statement

The authors declare that the research was conducted in the absence of any commercial or financial relationships that could be construed as a potential conflict of interest.
