# Supplementary material for: Global Epidemiology of Dengue Outbreaks in 1990–2015: A Systematic Review and Meta-Analysis
Source: Front Cell Infect Microbiol. 2017 Jul 12;7:317. doi: 10.3389/fcimb.2017.00317 (PMC5506197; doi:10.3389/fcimb.2017.00317)
Supplement: Supplementary file 1 [file Table1.DOCX]

***Supplementary material***

**Global Epidemiology of Dengue Outbreaks in 1990-2015: A Systematic Review and Meta-Analysis**

**1. Supplementary Figures and Tables**

**1.1. Supplementary Tables**

Supplementary Table 1. Details of 243 publications included in systematic review and meta-analysis

| PID | Publication year | First Author | Country | City | WHO region | Outbreak year |
| --- | --- | --- | --- | --- | --- | --- |
| 1 | 2016 | Zhao | China | Guangzhou | Western Pacific region | 2014 |
| 2 | 2016 | Xiao | China | Guangzhou | Western Pacific region | 2014 |
| 3 | 2016 | Wei | China | Taiwan | Western Pacific region | 2014 |
| 4 | 2016 | Villabona-Arenas | Brazil | Guaruja´, Sa˜o Paulo | Americas region | 2013 |
| 5 | 2016 | Vairo | United Republic of Tanzania | Dar es Salaam | African Region | 2014 |
| 6 | 2016 | Tun | Myanmar | Mandalay | South East Asia Region | 2013 |
| 7 | 2016 | Tittarelli | Argentina | Buenos Aires | Americas region | 2009 |
| 8 | 2016 | Thomas | United States of America | Texas | Americas region | 2013 |
| 9 | 2016 | Tazeen | India | New Delhi | South East Asia Region | 2014 |
| 10 | 2016 | Sun | China | Guangdong | Western Pacific region | 2014 |
| 11 | 2016 | Suleman | Pakistan | Swat and Mansehra | Eastern Mediterranean region | 2013 |
| 12 | 2016 | Succo | France | Nîmes | Europe Region | 2015 |
| 13 | 2016 | Siddiqui | India | Delhi | South East Asia Region | 2015 |
| 14 | 2016 | Saha | India | Kolkata | South East Asia Region | 2008;2009;2010;2011;2012 |
| 15 | 2016 | Quam | Japan | Tokyo | Western Pacific region | 2014 |
| 16 | 2016 | Phommanivong | Lao People's Democratic Republic | Champasak | Western Pacific region | 2013 |
| 17 | 2016 | Petitdemange | Gabon | Ogooue Lolo and Haut Ogooue provinces | African Region | 2010 |
| 18 | 2016 | Pessoa | Brazil | Tuparetama, Pernambuco | Americas region | 2015 |
| 19 | 2016 | Onoja | Nigeria | Ibadan | African Region | 2014 |
| 20 | 2016 | Massangaie | Mozambique | Pemba and Nampula | African Region | 2014 |
| 21 | 2016 | Mares-Guia | Brazil | Itaboraí, Rio de Janeiro | Americas region | 2013-2014 |
| 22 | 2016 | Lin | China | Guangzhou | Western Pacific region | 2014 |
| 23 | 2016 | Khurram | Pakistan | Rawalpindi | Eastern Mediterranean region | 2013 |
| 24 | 2016 | Johnston | United States of America | Hawaii | Americas region | 2015 |
| 25 | 2016 | Jones | Mexico | Yuma County, Arizona, and Sonora | Americas region | 2014 |
| 26 | 2016 | Huang | China | Guangdong | Western Pacific region | 2014 |
| 27 | 2016 | Haryanto | Indonesia | Jambi | South East Asia Region | 2015 |
| 28 | 2016 | Hapuarachchi | Singapore | / | Western Pacific region | 2013-2014 |
| 29 | 2016 | Carmo | Brazil | Marilia in northwest São Paulo State | Americas region | 2007 |
| 30 | 2016 | Dhanoa | Malaysia | Johor Bahru | Western Pacific region | 2014 |
| 31 | 2016 | Argolo | Brazil | Goiania | Americas region | 2012–2013 |
| 32 | 2016 | Cunha | Brazil | the state of Goiás | Americas region | 2013 |
| 33 | 2016 | Chetry | India | Pasighat, Arunachal Pradesh | South East Asia Region | 2015 |
| 34 | 2016 | Chen | China | Tainan, Taiwan, | Western Pacific region | 2015 |
| 35 | 2016 | Chen | China | Guangzhou | Western Pacific region | 2014 |
| 36 | 2016 | Chang | China | Taiwan | Western Pacific region | 2014 |
| 37 | 2016 | Castellanos | Colombian | Villa Nueva, Quibdó | Americas region | 2015 |
| 38 | 2016 | Buonora | Brazil | Rio de Janeiro | Americas region | 2013 |
| 39 | 2016 | Andrade | Brazil | Contagem, Minas Gerais | Americas region | 2013 |
| 40 | 2016 | Ali | Pakistan | Punjab; Khyber Pukhtunkhwa | Eastern Mediterranean region | 2011;2013 |
| 41 | 2016 | Acharyya | India | Howrah district | South East Asia Region | 2012-2013 |
| 42 | 2016 | Wang | China | Xishuangbanna and Dehong, Yunnan | Western Pacific region | 2013 |
| 43 | 2015 | Wang | China | Wenzhou and Wuhan | Western Pacific region | 2014 |
| 44 | 2015 | Wang | China | Zhongshan | Western Pacific region | 2013 |
| 45 | 2015 | Wang | China | Taiwan | Western Pacific region | 2014 |
| 46 | 2015 | Toan | Viet Nam | Hanoi | Western Pacific region | 2009 |
| 47 | 2015 | Le Viet | Viet Nam | Coastal island of Cat Ba | Western Pacific region | 2013 |
| 48 | 2015 | Takamatsu | Viet Nam | Central vietnam | Western Pacific region | 2013 |
| 49 | 2015 | Saswat | India | Odisha | South East Asia Region | 2013 |
| 50 | 2015 | Sasaki | Japan | / | Western Pacific region | 2014 |
| 51 | 2015 | Sahana | India | Bangalore, Karnataka | South East Asia Region | 2012 |
| 52 | 2015 | Kim Lien | Viet Nam | Hanoi | Western Pacific region | 2011 |
| 53 | 2015 | Pech Torres | Mexico | Yucatan | Americas region | 2011 |
| 54 | 2015 | Palanivel | India | Puducherry | South East Asia Region | 2012 |
| 55 | 2015 | Ng | Singapore and Malaysia | / | Western Pacific region | 2013 |
| 56 | 2015 | Leduc-Galindo | Mexico | Nuevo Leon state | Americas region | 2010 |
| 57 | 2015 | Kunwar | India | / | South East Asia Region | 2013 |
| 58 | 2015 | Guo | China | Xishuangbanna, Yunnan | Western Pacific region | 2013 |
| 59 | 2015 | Furuya | Japan | Tokyo’s Yoyogi Park | Western Pacific region | 2014 |
| 60 | 2015 | Ellis | Kenya | Mombasa | African Region | 2013 |
| 61 | 2015 | De Matos | Brazil | São Paulo State | Americas region | 2010 |
| 62 | 2015 | Vieira | Brazil | Sinop, Mato Grosso | Americas region | 2011-2012 |
| 63 | 2015 | Barde | India | tribal villages of Mandla district, Madhya Pradesh | South East Asia Region | 2013 |
| 64 | 2014 | Zhang | China | Xishuangbanna Dai Autonomous Prefecture,Yunnan | Western Pacific region | 2013 |
| 65 | 2014 | Williams | Peru | Loreto region | Americas region | 2010-2011 |
| 66 | 2014 | Wilder-Smith | Portugal | Madeira island | Europe Region | 2012 |
| 67 | 2014 | Vazquez | El Salvador | / | Americas region | 2012-2013 |
| 68 | 2014 | Tsegaye | Ethiopia | Dire Dawa town | African Region | 2013 |
| 69 | 2014 | Thomas | United States of America | Texas | Americas region | 2013 |
| 70 | 2014 | Stewart-Ibarra | Ecuador | Machala | Americas region | 2010 |
| 71 | 2014 | Sharp | Republic of the Marshall Islands | / | Western Pacific region | 2011-2012 |
| 72 | 2014 | Saqib | Pakistan | Lahore | Eastern Mediterranean region | 2011 |
| 73 | 2014 | Rezza | Yemen | Al Hudaydah | Eastern Mediterranean region | 2012 |
| 74 | 2014 | Pozo-Aguilar | Mexico | Chiapas | Americas region | 2009 |
| 75 | 2014 | Parreira | Angola | / | African Region | 2013 |
| 76 | 2014 | Pal | India | Kolkata | South East Asia Region | 2012 |
| 77 | 2014 | Ocwieja | Sri Lanka | / | South East Asia Region | 2012 |
| 78 | 2014 | Neeraja | India | Hyderabad, Andhra Pradesh State | South East Asia Region | 2011-2013 |
| 79 | 2014 | Naskar | India | West Bengal | South East Asia Region | 2012 |
| 80 | 2014 | Murad | Pakistan | Tehsil Chakaiser of District Shangla | Eastern Mediterranean region | 2008 |
| 81 | 2014 | Mogeni | Kenya | Mombasa County | African Region | 2013 |
| 82 | 2014 | Martins Vdo | Brazil | Amazonas | Americas region | 2011 |
| 83 | 2014 | Kyobe Bosa | Somalia | Mogadishu | Eastern Mediterranean region | 2011 |
| 84 | 2014 | Khan | India | Pasighat, East Siang district of Arunachal Pradesh (AP) state | South East Asia Region | 2012 |
| 85 | 2014 | Jindal | India | Malwa region of Punjab | South East Asia Region | 2011 |
| 86 | 2014 | Hunsperger | Somalia | / | Eastern Mediterranean region | 2011 |
| 87 | 2014 | Huang | China | Henan | Western Pacific region | 2013 |
| 88 | 2014 | Figueiredo | Brazil | Piaui | Americas region | 2006;2007 |
| 89 | 2014 | Faye | Senegal | / | African Region | 2009 |
| 90 | 2014 | Fariz-Safhan | Malaysia | Pahang | Western Pacific region | 2005 |
| 91 | 2014 | Cavalcanti | Brazil | Fortaleza in the state of Ceará | Americas region | 2011 |
| 92 | 2014 | Carvalho | Brazil | Cruzeiro, São Paulo | Americas region | 2006;2011 |
| 93 | 2014 | Deligny | France | French West Indies | Europe Region | 2010 |
| 94 | 2014 | Chen | China | Taiwan | Western Pacific region | 2002-2003 |
| 95 | 2014 | Chatterjee | India | West Bengal | South East Asia Region | 2012 |
| 96 | 2014 | Assir | Pakistan | Lahore | Eastern Mediterranean region | 2012 |
| 97 | 2014 | Assir | Pakistan | Lahore | Eastern Mediterranean region | 2011 |
| 98 | 2014 | Allonso | Brazil | Rio de Janeiro | Americas region | 2012 |
| 99 | 2013 | VinodKumar | India | Davangere, Karnataka | South East Asia Region | 2011-2012 |
| 100 | 2013 | Thangaratham | India | Tirupur | South East Asia Region | 2005 |
| 101 | 2013 | Taulung | Federated States of Micronesia | Kosrae | Western Pacific region | 2012-2013 |
| 102 | 2013 | Schwartz | Angola | Luanda | African Region | 2013 |
| 103 | 2013 | Pun | Nepal | Butwal city | South East Asia Region | 2010 |
| 104 | 2013 | Neeraja | India | Southern India | South East Asia Region | 2007 |
| 105 | 2013 | Madani | Yemen | Al-Mukalla, Hadrahmout | Eastern Mediterranean region | 2010 |
| 106 | 2013 | Lorenzi | United States of America | Ponce, Puerto Rico | Americas region | 2009 |
| 107 | 2013 | Khan | India | Moreh township, Manipur | South East Asia Region | 2007 |
| 108 | 2013 | Kalappanvar | India | Davangere, Karnataka | South East Asia Region | 2009-2010 |
| 109 | 2013 | Ho | China | Southern Taiwan | Western Pacific region | 2007 |
| 110 | 2013 | Goyal | India | Bathinda, Punjab | South East Asia Region | 2011 |
| 111 | 2013 | Fahri | Indonesia | Semarang | South East Asia Region | 2011-2012 |
| 112 | 2013 | Dhar | India | Uttarakhand, Dehradun | South East Asia Region | 2010 |
| 113 | 2013 | Ahmed | Pakistan | Lahore | Eastern Mediterranean region | 2011 |
| 114 | 2013 | Santiago | Federated States of Micronesia | Kosrae | Western Pacific region | 2012-2013 |
| 115 | 2012 | Ujwala | India | Miraj | South East Asia Region | 2012 |
| 116 | 2012 | Sun | China | Zhejiang | Western Pacific region | 2009 |
| 117 | 2013 | Sousa | Portugal | Madeira | Europe Region | 2012 |
| 118 | 2012 | Peng | China | Dongguang | Western Pacific region | 2010 |
| 119 | 2012 | Nkoghe | Gabon | / | African Region | 2010 |
| 120 | 2012 | Jing | China | Guangzhou, Jingtai Street | Western Pacific region | 2010 |
| 121 | 2011 | Vedpathak | India | Ambajogai Mandal, Beed, Maharashtra | South East Asia Region | 2006 |
| 122 | 2011 | Mohammad | Bangladesh | Dhaka | South East Asia Region | 2000-2001 |
| 123 | 2011 | Malik | Sudan | Port | Eastern Mediterranean region | 2004-2005 |
| 124 | 2011 | Giraldo | Brazil | Rio de Janeiro | Americas region | 2007-2008 |
| 125 | 2010 | Mohammed | United States of America | United States Virgin Islands: St. Croix | Americas region | 2005 |
| 126 | 2010 | Kulkarni | India | Jaipur | South East Asia Region | 2005 |
| 127 | 2010 | Humayoun | Pakistan | Punjab | Eastern Mediterranean region | 2008 |
| 128 | 2009 | Tomashek | United States of America | Puerto Rico | Americas region | 2007 |
| 129 | 2009 | Suharti | Indonesia | Semarang | South East Asia Region | 1995-1996 |
| 130 | 2009 | Riaz | Pakistan | Karachi | Eastern Mediterranean region | 2006 |
| 131 | 2009 | Meynard | France | Maripasoula, France Guiana | Americas region | 2005-2006 |
| 132 | 2009 | Kularatne | Sri Lanka | Kandy | South East Asia Region | 2006–2007 |
| 133 | 2009 | De Araujo | Brazil | de Janeiro | Americas region | 2002 |
| 134 | 2008 | Thomas | France | Martinique | Europe Region | 2005-2006 |
| 135 | 2008 | Tang | China | Guangzhou | Western Pacific region | 2006 |
| 136 | 2008 | Tang | Pakistan | / | Eastern Mediterranean region | 2006 |
| 137 | 2008 | Ratsitorahina | Madagascar | Toamasina | African Region | 2006 |
| 138 | 2008 | Rai | India | Delhi | South East Asia Region | 2006 |
| 139 | 2008 | Passons | Brazil | Rio de Janeiro | Americas region | 2001-2002 |
| 140 | 2008 | Liu | China | Kaohsiung, Taiwan | Western Pacific region | 2002 |
| 141 | 2008 | Lee | Singapore | Singapore | Western Pacific region | 2004 |
| 142 | 2008 | Kuo | China | Taiwan | Western Pacific region | 2002 |
| 143 | 2008 | Koh | Singapore | / | Western Pacific region | 2005 |
| 144 | 2008 | Khan | Saudi Arabia | Makkah | Eastern Mediterranean region | 2004 |
| 145 | 2008 | Khan | Pakistan | Karachi | Eastern Mediterranean region | 2006 |
| 146 | 2008 | Ghani | Pakistan | Sindh | Eastern Mediterranean region | 2006 |
| 147 | 2008 | Dutta | India | Kolkata | South East Asia Region | 2005 |
| 148 | 2008 | Chandralekha | India | north India | South East Asia Region | 2006 |
| 149 | 2008 | Bharaj | India | New Delhi | South East Asia Region | 2006 |
| 150 | 2008 | Batuah | India | Moreh in Manipur | South East Asia Region | 2007 |
| 151 | 2008 | Ahmed | Pakistan | Karachi | Eastern Mediterranean region | 2006 |
| 152 | 2007 | Zhang | China | Guangzhou | Western Pacific region | 2002;2003;2006 |
| 153 | 2007 | Xu | China | Ningbo | Western Pacific region | 2004 |
| 154 | 2007 | Wang | China | southern Taiwan | Western Pacific region | 2002-2003 |
| 155 | 2007 | Thomas | India | Delhi, Ludhiana | South East Asia Region | 2003 |
| 156 | 2007 | Seet | Singapore | Singapore | Western Pacific region | 2005 |
| 157 | 2007 | Samuel | India | Tamil Nadu | South East Asia Region | 2003 |
| 158 | 2007 | Mourao | Brazil | Manaus, Amazon | Americas region | 2001 |
| 159 | 2007 | Levi | Brazil | Goiania | Americas region | 2005 |
| 160 | 2007 | Kularatne | Sri Lanka | Kandy | South East Asia Region | 2005 |
| 161 | 2007 | Khan | Pakistan | karachi | Eastern Mediterranean region | 2006 |
| 162 | 2007 | Chowell | Mexico | Colima | Americas region | 2002 |
| 163 | 2007 | Wang | China | Kaohsiung, Taiwan | Western Pacific region | 2002 |
| 164 | 2006 | Suwandono | Indonesia | Jakarta | South East Asia Region | 2004 |
| 165 | 2006 | Lee | China | Kaohsiung, Taiwan | Western Pacific region | 2002 |
| 166 | 2006 | Kapoor | India | East Indian | South East Asia Region | 2003 |
| 167 | 2006 | Hoti | India | Pondicherry | South East Asia Region | 2003-2004 |
| 168 | 2006 | Hayes | United States of America | Nahiku, Hawaii | Americas region | 2001 |
| 169 | 2006 | Hati | India | West Bengal state | South East Asia Region | 2005 |
| 170 | 2006 | Hanna | Australia | Cairns; Townsville; The Torres Strait | Western Pacific region | 2003-2004 |
| 171 | 2006 | Dash | India | Delhi | South East Asia Region | 2004 |
| 172 | 2006 | Dar | India | Delhi | South East Asia Region | 2003 |
| 173 | 2006 | Chua | Malaysia | Klang | Western Pacific region | 2004-2005 |
| 174 | 2006 | Cheah | Malaysia | Lundu District in Sarawak | Western Pacific region | 1999 |
| 175 | 2005 | Singh | India | Delhi | South East Asia Region | 2003 |
| 176 | 2005 | Seet | Singapore | / | Western Pacific region | 2002 |
| 177 | 2005 | Rodriguez-Roche | Cuba | Havana | Americas region | 2000;2001-2002 |
| 178 | 2005 | Rodriguez-Roche | Cuba | Santiago de Cuba | Americas region | 1997 |
| 179 | 2005 | Ratho | India | Chandigarh | South East Asia Region | 2002 |
| 180 | 2005 | Peyrefitte | France | Saint Martin island, French West Indies | Europe Region | 2003-2004 |
| 181 | 2005 | Itha | India | Uttar Pradesh | South East Asia Region | 2003 |
| 182 | 2005 | Gupta | India | Delhi | South East Asia Region | 2003 |
| 183 | 2005 | Espinoza-Gómez | Mexico | Colima | Americas region | 2002 |
| 184 | 2005 | Effler | United States of America | Hawaii | Americas region | 2001-2002 |
| 185 | 2005 | Durand | Federated States of Micronesia | Yap state | Western Pacific region | 2004 |
| 186 | 2005 | Dash | India | Gwalior, Madhya Pradesh and Delhi, India | South East Asia Region | 2003 |
| 187 | 2005 | Chakravarti | India | Delhi | South East Asia Region | 2003 |
| 188 | 2004 | Buchy | Viet Nam | Central Vietnam | Western Pacific region | 2001-2002 |
| 189 | 2004 | Wiwanitkit | Thailand | Surin Province | South East Asia Region | 2001 |
| 190 | 2004 | Wichmann | Thailand | Chonburi | South East Asia Region | 2001 |
| 191 | 2004 | Wagatsuma | Bangladesh | Dhaka | South East Asia Region | 2001 |
| 192 | 2004 | Tran | France | Iracoubo, French Guiana | Americas region | 2001 |
| 193 | 2004 | Shah | India | Mumbai | South East Asia Region | 2003 |
| 194 | 2004 | Pervin | Bangladesh | Dhaka | South East Asia Region | 2000 |
| 195 | 2004 | Lorono-Pino | Mexico | Yucatan State | Americas region | 2002 |
| 196 | 2004 | Lai | China | Kaohsiung, Taiwan | Western Pacific region | 2001 |
| 197 | 2004 | Kumar | India | Delhi | South East Asia Region | 2003 |
| 198 | 2004 | Chao | China | Taiwan | Western Pacific region | 1998 |
| 199 | 2004 | Chadee | Trinidad and Tobago | / | Americas region | 1998 |
| 200 | 2003 | Wang | China | Taiwan | Western Pacific region | 2000 |
| 201 | 2003 | Wang | China | Taiwan | Western Pacific region | 1998 |
| 202 | 2003 | Tuntaprasart | Thailand | Mueang district, Ratchaburi | South East Asia Region | 2000-2001 |
| 203 | 2003 | Sukri | Indonesia | Merauke, Papua | South East Asia Region | 2001 |
| 204 | 2003 | Reiter | United States of America and Mexico | Texas, America and Taumalipas, Mexico | Americas region | 1999 |
| 205 | 2003 | Perret | Chile | Easter Island | Americas region | 2002 |
| 206 | 2003 | Patumanond | Thailand. | Uttaradit | South East Asia Region | 1993;1998;2001 |
| 207 | 2003 | Narayanan | India | Chennai | South East Asia Region | 2001 |
| 208 | 2003 | Liu | China | Taiwan | Western Pacific region | 2001 |
| 209 | 2003 | Kabilan | India | Chennai, Tamil Nadu | South East Asia Region | 2001 |
| 210 | 2003 | Hayes | El Salvador | / | Americas region | 2000 |
| 211 | 2003 | Hanna | Australia | North Queensland | Western Pacific region | 2002 |
| 212 | 2003 | Ashford | Palau | / | Western Pacific region | 1995 |
| 213 | 2002 | Rahman | Bangladesh | Dhaka | South East Asia Region | 2000 |
| 214 | 2002 | Parida | India | Gwalior | South East Asia Region | 2001 |
| 215 | 2002 | Hills | Australia | Townsville, North Queensland |  | 2001 |
| 216 | 2002 | Guzmán | Cuba | Santiago de Cuba | Americas region | 1997 |
| 217 | 2002 | Aziz | Bangladesh | / | South East Asia Region | 2000 |
| 218 | 2001 | Yunus | Bangladesh | / | South East Asia Region | 2000 |
| 219 | 2001 | Rigau-Perez | United States of America | Villalba, Puerto Rico | Americas region | 1995 |
| 220 | 2001 | Lin | China | Taiwan | Western Pacific region | 1998-1999 |
| 221 | 2001 | Heukelbach | Brazil | Fortaleza | Americas region | 1999 |
| 222 | 2001 | Ahmed | Bangladesh | Chittagong | South East Asia Region | 2000 |
| 223 | 2000 | Murgue | French Polynesia (France) | / | Western Pacific region | 1996-1997 |
| 224 | 2000 | Lyerla | British Virgin Islands | Tortola | Americas region | 1995 |
| 225 | 2000 | Guzmán | Cuba | Santiago de Cuba | Americas region | 1997 |
| 226 | 1999 | Wali | India | / | South East Asia Region | 1996 |
| 227 | 1999 | Guzman | Cuba | / | Americas region | 1997 |
| 228 | 1999 | Dar | India | Delhi | South East Asia Region | 1996 |
| 229 | 1998 | Savage | Federated States of Micronesia | Yap state | Western Pacific region | 1995 |
| 230 | 1998 | Paul | Pakistan | Baluchistan | Eastern Mediterranean region | 1995 |
| 231 | 1998 | Morrison | United States of America | Florida, Puerto Rico | Americas region | 1991-1992 |
| 232 | 1998 | Kouri | Cuba | Santiago de | Americas region | 1997 |
| 233 | 1998 | Hanna | Australia | Queensland | Western Pacific region | Torres Strait 1996; Cairn suburb 1997 |
| 234 | 1998 | Deparis | French Polynesia (France) | / | Western Pacific region | 1996 |
| 235 | 1998 | Anuradha | India | Delhi | South East Asia Region | 1996 |
| 236 | /1997 | Richards | Indonesia | Jayapura, the provincial capital of hian Jaya, | South East Asia Region | 1993-1994 |
| 237 | 1997 | Griffiths | Australia | Cairns | Western Pacific region | 1996 |
| 238 | 1996 | Murray-Smith | Australia | Charters Towers, Queensland | Western Pacific region | 1993 |
| 239 | 1995 | Vasconcelos | Brazil | Ceara | Americas region | 1994 |
| 240 | 1995 | Rodriguez-Figueroa | United States of America | Yanes, Puerto Rico | Americas region | 1991 |
| 241 | 1995 | Nogueira | Brazil | Bahia | Americas region | 1994 |
| 242 | 1994 | Reynes | France | French Guiana | Americas Region | 1991-1992 |
| 243 | 1992 | Phillips | Peru | Loreto, Peruvian Amazon | Americas region | 1990 |

*PID*, publication identification.

Supplementary Table 2. Articles included in the meta-analysis of epidemiologic factors

| Variables | No. studies meta-analyzed | PID of papers included |
| --- | --- | --- |
| Mean age | 96 | 1,3,6,7,8,9,11,18,23,24,29,30,31,34,35,39,41,42,43,46,47,52,53,56,60,65,71,73,75,80,82,83,84,88,89,90,91,93,95,96,97,98,100,102,103,107,108,111,113,119,120,125,128,129,130,132,134,139,142,144,145,149,151,152,156,159,161,162,164,165,166,168,172,175,181,184,185,190,191,193,194,198,201,203,208,210,217,219,220,222,223,226,233,234,235,239 |
| Male sex | 146 | 1,2,3,5,6,7,8,9,10,11,12,18,19,20,21,22,23,24,25,26,27,29,30,31,34,35,39,40,41,42,43,44,46,47,49,51,52,53,54,56,57,60,61,63,65,71,72,74,75,78,79,80,82,83,84,87,88,89,90,91,93,94,95,96,97,98,99,100,102,103,104,108,109,113,116,119,120,121,122,123,124,125,126,127,128,129,130,132,134,135,138,139,140,141,142,144,145,147,148,149,150,151,152,153,155,156,160,161,162,163,165,166,168,169,172,175,181,182,184,185,188,190,191,196,197,198,199,201,208,217,218,219,220,221,222,223,224,226,227,228,231,235,238,239,240,243 |
| DHF | 107 | 3,8,20,23,25,27,45,53,54,56,67,70,72,78,89,90,91,93,94,95,97,100,102,108,109,111,112,113,116,122,123,124,125,126,127,128,129,130,133,134,135,138,139,140,141,142,143,144,148,149,150,151,152,154,155,156,158,160,161,162,164,165,166,167,176,177,178,181,183,184,185,189,190,192,193,194,195,196,197,198,199,201,202,203,205,207,208,209,213,215,216,218,220,222,223,225,226,227,228,232,233,234,235,236,239,242,243 |
| Secondary infection | 35 | 1,3,6,7,13,27,30,33,37,38,67,71,90,93,98,100,104,111,112,125,160,163,164,169,178,190,191,194,198,201,210,213,216,223,232 |
| Fatal cases | 72 | 1,2,3,10,20,22,23,24,34,41,43,45,51,54,55,56,63,64,71,72,75,78,79,89,90,98,108,113,114,123,128,133,134,138,140,142,143,146,148,151,161,165,175,177,181,183,184,185,189,190,191,193,195,207,210,213,216,218,222,223,225,226,227,228,229,232,233,234,235,239,242,243 |

*PID*, publication identification.

Supplementary Table 3. Articles included in the meta-analysis of risk factors associated with dengue infection

| Variables | No. studies meta-analyzed | Meta-analysis, pooled data (95% CI) | *P-*values | Total cases/  total controls | PID of papers included |
| --- | --- | --- | --- | --- | --- |
| Gender | 16 | 1.10 (1.01 – 1.20) | 0.030 | 6581/5835 | 5,11,20,21,29,60,63,71,106,125,128,139,147,184,188,240 |
| Age* | 3 | 1.36 (-2.89 – 5.60) | 0.530 | 822/255 | 11,29,234 |
| Illiteracy | 2 | 0.64 (0.17 – 2.36) | 0.506 | 256/276 | 20,80 |
| Uncover water container | 3 | 1.65 (1.15 – 2.37) | 0.007 | 263/290 | 46,80,221 |
| Empty receptacles in house/garden/courtyard | 2 | 3.43 (0.98 – 12.00) | 0.054 | 190/217 | 80,221 |
| Did not use mosquito repellent | 3 | 0.92 (0.26 – 3.28) | 0.897 | 281/1420 | 60,191,240 |
| Used bed net | 3 | 1.35 (0.56 – 3.26) | 0.504 | 369/1726 | 5,60,240 |
| Had screens on windows or door | 3 | 0.27 (0.04 - 1.95) | 0.187 | 466/1562 | 60,168,238 |
| Used air conditioning | 2 | 0.66 (0.24 – 1.84) | 0.424 | 449/1567 | 60,204 |
| Animals on property | 3 | 1.29 (0.38 – 4.00) | 0.672 | 218/261 | 80,168,221 |
| Fever | 8 | 1.39 (0.57 – 3.43) | 0.472 | 1724/2932 | 21,60,106,109,139,164,184,234 |
| Myalgia | 7 | 1.38 (1.08 – 1.75) | 0.009 | 1535/1698 | 20,21,38,109,139,164,184 |
| Headache | 10 | 1.55 (0.93 – 2.60) | 0.095 | 1870/1929 | 20,21,38,106,109,139,164,184,188,234 |
| Itching | 2 | 1.35 (0.22 – 8.36) | 0.746 | 829/194 | 109,139 |
| Chill | 2 | 1.67 (1.13 – 2.46) | 0.010 | 222/1193 | 20,184 |
| Arthralgia | 7 | 1.31 (0.97 – 1.78) | 0.081 | 1206/1766 | 20,21,38,106,139,184,234 |
| Rash | 5 | 2.77 (1.74 – 4.42) | <0.001 | 785/1521 | 20,21,106,109,184 |
| Eye/retro-orbital pain | 8 | 1.27 (1.04 – 1.55) | 0.019 | 1298/1713 | 20,21,38,106,139,164,184,188 |
| Nausea | 3 | 1.25 (0.82 – 1.88) | 0.292 | 375/270 | 147,164,188 |
| Vomiting | 5 | 0.80 (0.40 – 1.61) | 0.530 | 1024/452 | 139,147,164,188,234 |
| Persistent vomiting | 5 | 1.42 (0.84 – 2.42) | 0.194 | 1356/2329 | 20,38,60,71,106 |
| Diarrhea | 5 | 1.25 (0.97 – 1.60) | 0.080 | 950/1511 | 20,21,109,184,234 |
| Sore throat | 3 | 0.47 (0.23 – 0.95) | 0.037 | 333/1244 | 106,164,184 |
| Nasal congestion | 2 | 0.35 (0.14 – 0.89) | 0.026 | 153/1212 | 106,184 |
| Cough | 2 | 0.26 (0.12 – 0.58) | 0.001 | 153/1212 | 106,184 |
| Petechiae | 5 | 1.95 (1.24 – 3.07) | 0.004 | 972/2623 | 21,60,109,184,188 |
| Abdominal pain | 8 | 1.12 (0.78 – 1.59) | 0.533 | 2022/1314 | 20,71,106,139,147,164,188,234 |
| Exanthema | 2 | 1.97 (1.23 – 3.17) | 0.005 | 601/257 | 38,139 |
| Any blood manifestation | 10 | 1.27 (0.88 – 1.85) | 0.207 | 4966/4199 | 20,21,38,60,128,139,164,184,188,234 |
| Hematuria | 2 | 0.60 (0.14 – 2.59) | 0.493 | 575/1180 | 139,184 |
| Lethargy | 2 | 1.78 (1.02 – 3.08) | 0.041 | 1015/834 | 38,71 |
| Liver enlargement | 3 | 1.27 (0.48 – 3.40) | 0.632 | 1046/946 | 38,71,106 |
| Lymphadenopathy | 2 | 2.21 (1.08 – 4.54) | 0.030 | 649/182 | 139,234 |
| Thrombocytopenia | 3 | 3.31 (2.17 – 5.03) | <0.001 | 524/311 | 38,164,234 |
| Leukopenia | 3 | 4.42 (1.75 – 11.20) | 0.002 | 524/311 | 38,164,234 |
| Conjunctival injection | 2 | 1.51 (1.01 – 2.26) | 0.045 | 256/194 | 38,188 |
| Positive tourniquet test | 4 | 4.86 (1.07 – 22.03) | 0.040 | 695/275 | 106,109,164,188 |
| Hospitalized | 5 | 0.95 (0.54 – 1.68) | 0.859 | 4592/4346 | 20,60,71,128,184 |

*Mean difference and its 95% confidence interval; *PID*, publication identification.

Supplementary Table 4. Articles included in the meta-analysis of risk factors associated with DHF/DSS

| Variables | No. studies meta-analyzed | Meta-analysis, pooled data (95% CI) | *P*-values | Total cases/  total controls | | PID of papers included |
| --- | --- | --- | --- | --- | --- | --- |
| Gender | 14 | 1.15 (0.98 – 1.36) | 0.095 | 1115/3546 | 27,90,94,113,127,140,141,142,154,161,163,165,201,220 | |
| Age* | 8 | 2.34 (-1.48 – 6.17) | 0.230 | 727/1106 | 90,113,140,154,161,165,201,220 | |
| Diabetes mellitus | 3 | 2.31 (1.58 – 3.38) | <0.001 | 423/874 | 140,142,165 | |
| Hypertension | 3 | 1.36 (0.80 – 2.31) | 0.256 | 423/874 | 140,142,165 | |
| Hypotension | 4 | 6.18 (1.61 – 23.71) | 0.008 | 289/2209 | 78,130,141,222 | |
| HBV/HCV infection | 2 | 0.98 (0.58 – 1.65) | 0.940 | 316/462 | 140,165 | |
| Renal insufficiency | 2 | 5.26 (1.77 – 15.64) | 0.003 | 316/462 | 140,165 | |
| Fever | 5 | 0.41 (0.15 – 1.13) | 0.083 | 442/781 | 72, 140,161,167,195 | |
| Myalgia | 6 | 1.14 (0.66 – 1.99) | 0.642 | 708/1253 | 78,112,122,126,167,195 | |
| Headache | 12 | 0.76 (0.47 – 1.24) | 0.267 | 1356/2408 | 72,78,112,113,122,126,130,140,161,195,222,236 | |
| Body ache | 2 | 0.60 (0.36 – 0.99) | 0.048 | 287/525 | 113,161 | |
| Arthralgia | 5 | 1.57 (1.09 – 2.26) | 0.015 | 754/1087 | 112,122,126,140,195 | |
| Rash | 11 | 1.18 (0.86 – 1.61) | 0.301 | 1312/3992 | 72,78,112,122,126,130,140,141,142,161,222 | |
| Macular rash | 2 | 0.35 (0.03 – 4.14) | 0.404 | 215/59 | 122,155 | |
| Maculopapular rash | 3 | 0.32 (0.02 – 5.54) | 0.427 | 238/66 | 122,155,236 | |
| Retro-orbital pain | 4 | 1.35 (0.77 -2.38) | 0.297 | 343/484 | 112,122,130,222 | |
| Eye pain | 2 | 0.62 (0.12 – 3.31) | 0.572 | 151/51 | 161,236 | |
| Nausea | 4 | 1.01 (0.42 – 2.41) | 0.982 | 667/892 | 112,122,126,140 | |
| Vomiting | 9 | 1.16 (0.68 – 1.97) | 0.584 | 1032/2000 | 72,78,112,113,122,126,140,141,222,236 | |
| Diarrhea | 7 | 1.37 (0.71 – 2.66) | 0.350 | 578/585 | 112,122,130,140,161,222,236 | |
| Sore throat | 2 | 0.85 (0.43 – 1.67) | 0.639 | 132/176 | 130,236 | |
| Epistaxis | 6 | 2.03 (1.57 – 2.63) | <0.001 | 767/849 | 122,126,140,195,222,236 | |
| Cough | 5 | 1.10 (0.73 – 1.65) | 0.647 | 724/648 | 122,126,161,222,236 | |
| Petechiae | 6 | 1.74 (1.24 – 2.44) | 0.001 | 516/723 | 122,140,142,155,195,236 | |
| Purpura | 2 | 3.15 (2.04 – 4.87) | <0.001 | 291/499 | 113,122 | |
| Ecchymosis | 3 | 2.75 (1.20 – 6.31) | 0.017 | 302/254 | 122,155,195 | |
| Facial flushing | 2 | 0.33 (0.01 – 11.59) | 0.538 | 192/210 | 130,155 | |
| Altered Sensorium | 2 | 0.96 (0.09 – 10.62) | 0.973 | 411/712 | 78,126 | |
| Anorexia | 3 | 0.85 (0.32 – 2.30) | 0.747 | 611/621 | 122,126,140 | |
| Jaundice | 2 | 2.63 (0.73 – 9.48) | 0.139 | 441/579 | 126,222 | |
| Abdominal pain | 10 | 1.58 (0.97 – 2.57) | 0.066 | 1302/3772 | 72,90,112,113,122,126,140,141,161,236 | |
| Abdominal distension | 2 | 2.73 (0.33 -22.43) | 0.351 | 178/44 | 122,222 | |
| Any blood manifestation | 9 | 9.57 (4.78 – 19.15) | <0.001 | 1091/2210 | 72,78,112,126,130,142,167,190,222 | |
| Gum bleeding | 6 | 3.78 (0.72 – 19.79) | 0.116 | 798/2509 | 122,126,140,141,222,236 | |
| Melena | 5 | 5.67 (1.73 – 18.63) | 0.004 | 372/296 | 122,140,195,222,236 | |
| Hematuria | 6 | 1.55 (1.04 – 2.31) | 0.031 | 479/708 | 122,140,142,195,222,236 | |
| Hematemesis | 4 | 3.68 (1.42 – 9.52) | 0.007 | 288/246 | 122,195,222,236 | |
| Hemoptysis | 2 | 4.54 (0.87 – 23.69) | 0.073 | 216/68 | 122,140 | |
| Shock | 3 | 2.63 (1.01 – 6.84) | 0.048 | 152/133 | 140,167,222 | |
| Convulsions | 3 | 2.64 (0.69 – 10.05) | 0.155 | 91/90 | 167,222,236 | |
| Tachycardia | 2 | 2.89 (0.25 – 32.93) | 0.394 | 155/195 | 130,222 | |
| Hyponatremia | 2 | 1.14 (0.41 – 3.16) | 0.801 | 216/581 | 130,142 | |
| Proteinuria | 2 | 2.72 (2.04 – 3.63) | <0.001 | 339/824 | 142,165 | |
| Hepatomegaly | 7 | 6.39 (4.93 – 8.30) | <0.001 | 813/888 | 90,122,126,130,167,222,236 | |
| Splenomegaly | 3 | 6.80 (4.49 – 10.29) | <0.001 | 573/597 | 122,126,222 | |
| Thrombocytopenia | 3 | 7.02 (0.38 – 128.99) | 0.190 | 260/220 | 130,161,236 | |
| Pleural effusion | 10 | 12.44 (7.07 – 21.91) | <0.001 | 713/902 | 78,90,94,113,122,140,154,161,222,236 | |
| Ascites | 10 | 13.91 (8.03 – 24.11) | <0.001 | 719/766 | 90,94,113,122,140,154,161,181,222,236 | |
| APTT prolongation | 2 | 5.73 (0.88 – 37.48) | 0.068 | 352/460 | 165,208 | |
| Positive tourniquet test | 5 | 4.15 (1.55 – 11.08) | 0.005 | 506/424 | 122,167,190,195,222 | |
| Leukopenia | 6 | 0.95 (0.57 – 1.59) | 0.845 | 475/717 | 78,112,130,161,208,222 | |
| Leukocytosis | 2 | 7.11 (0.05 – 1047.20) | 0.440 | 166/74 | 208,222 | |
| Monocytosis | 2 | 1.70 (0.47 – 6.05) | 0.416 | 248/92 | 161,208 | |
| Raised ALT | 6 | 2.31 (1.82 – 2.93) | <0.001 | 1006/1249 | 126,130,161,165,181,208 | |
| Raised AST | 5 | 2.60 (1.05 – 6.47) | 0.039 | 774/837 | 126,130,161,181,208 | |
| Secondary infection | 9 | 1.86 (1.46 – 2.37) | <0.001 | 670/1221 | 27,90,140, 142,164, 165,178,201,213 | |
| Death | 3 | 11.46 (4.16 – 31.56) | <0.001 | 519/937 | 113,161,165 | |

*Mean difference and its 95% confidence interval; *PID*, publication identification.

Supplementary Table 5. Articles included in the meta-analysis of risk factors associated with secondary infection cases

| Variables | No. studies meta-analyzed | Meta-analysis, pooled data (95% CI) | *P*-values | Total cases/  total controls | PID of papers included |
| --- | --- | --- | --- | --- | --- |
| Gender (male) | 2 | 1.34 (0.82 – 2.18) | 0.241 | 91/237 | 7,134 |
| Platelet count < 100 × 10^9^/L | 2 | 4.11 (1.64 – 10.31) | 0.003 | 90/124 | 134,194 |

*PID*, publication identification.

Supplementary Table 6. Articles included in the meta-analysis of risk factors associated with Death

| Variables | No. studies meta-analyzed | Meta-analysis, pooled data (95% CI) | *P*-values | Total cases/  total controls | PID of papers included |
| --- | --- | --- | --- | --- | --- |
| Gender | 4 | 0.79 (0.49 – 1.28) | 0.336 | 86/867 | 3,34,140,142 |
| Diabetes mellitus | 4 | 2.53 (1.51 – 4.24) | <0.001 | 74/360 | 3,34,140,142 |
| Hypertension | 4 | 2.36 (1.37 – 4.07) | 0.002 | 74/360 | 3,34,140,142 |
| Cardiovascular disease | 2 | 1.86 (0.68 – 5.10) | 0.227 | 32/623 | 3,142 |
| Cancers | 2 | 1.52 (0.56 – 4.16) | 0.413 | 45/617 | 34,142 |
| Fever | 3 | 1.14 (0.47 – 2.71) | 0.769 | 74/360 | 3,34,140 |
| Myalgia | 3 | 1.14 (0.63 – 2.06) | 0.665 | 74/360 | 3,34,140 |
| Headache | 2 | 0.46 (0.22 – 0.95) | 0.037 | 41/250 | 3,140 |
| Bone pain | 2 | 0.44 (0.19 – 1.03) | 0.057 | 41/250 | 3,140 |
| Arthralgia | 2 | 1.09 (0.48 – 2.46) | 0.836 | 41/250 | 3,140 |
| Rash | 3 | 0.80 (0.39 – 1.62) | 0.539 | 74/360 | 3,34,140 |
| Vomiting | 2 | 1.71 (0.85 – 3.44) | 0.133 | 41/250 | 3,140 |
| Diarrhea | 3 | 1.61 (0.85 – 3.05) | 0.144 | 74/360 | 3,34,140 |
| Anorexia | 2 | 0.98 (0.52 – 1.84) | 0.950 | 54/244 | 34,140 |
| Abdominal pain | 3 | 1.27 (0.72 – 2.24) | 0.409 | 74/360 | 3,34,140 |
| Gastrointestinal bleeding | 2 | 3.97 (0.40 – 39.75) | 0.240 | 54/244 | 34,140 |
| Gum bleeding | 2 | 0.95 (0.37 – 2.40) | 0.914 | 54/244 | 34,140 |
| Hematuria | 2 | 1.33 (0.61 – 2.89) | 0.472 | 54/244 | 34,140 |
| Shock | 2 | 308.09 (42.56 – 2230.53) | <0.001 | 26/201 | 140,222 |
| Pleural effusion | 2 | 2.00 (0.83 – 4.84) | 0.123 | 26/201 | 140,222 |
| WBC count ≥10000/ul | 2 | 1.75 (0.41 – 7.52) | 0.451 | 41/250 | 3,140 |
| Secondary infection | 2 | 1.04 (0.44 – 2.50) | 0.929 | 41/250 | 3,140 |

*PID*, publication identification.

Supplementary Table 7. Articles included in the meta-analysis of symptoms and signs

| Variables | No. studies meta-analyzed | PID of papers included |
| --- | --- | --- |
| Fever | 88 | 12,21,22,25,26,30,34,43,51,53,54,57,60,63,72,73,75,76,78,80,84,89,95,96,98,99,100,102,104,105,106,108,111,112,113,118,119,120,121,122,123,124,125,127,132,138,139,144,147,148,149,151,152,153,155,156,161,162,164,165,166,167,175,176,181,184,186,189,190,191,193,194,195,196,208,209,212,213,217,218,224,226,227,234,235,236,242,243 |
| Headache | 83 | 12,20,21,25,26,30,38,43,51,54,57,63,72,73,74,75,78,80,82,84,89,91,95,96,100,102,104,105,106,107,109,111,112,113,115,118,119,120,121,122,124,125,126,127,129,130,132,138,139,144,148,151,152,153,156,160,161,162,164,165,166,175,176,184,188,190,191,194,195,196,208,212,213,217,218,222,224,226,227,234,236,242,243 |
| Malaise | 9 | 12,105,120,139,144,165,226,227,243 |
| Retro-orbital pain | 38 | 12,20,21,38,57,74,75,84,91,96,98,99,100,105,108,111,112,120,121,122,124,125,127,129,130,139,148,151,162,164,165,166,184,188,194,196,208,222 |
| Dizziness | 9 | 43,60,74,84,91,100,144,208,243 |
| Chills | 14 | 20,30,43,96,125,130,132,156,162,176,184,196,224,243 |
| Myalgia | 65 | 20,21,25,26,30,34,38,43,51,54,73,74,75,78,79,80,82,84,89,91,95,96,97,98,100,105,109,111,118,119,120,121,122,126,127,132,138,139,144,148,151,152,153,155,156,160,162,164,165,166,167,184,188,190,191,194,195,196,208,212,213,224,235,242,243 |
| Body-ache | 13 | 22,57,63,106,113,115,125,146,161,175,181,217,218 |
| Arthralgia | 53 | 20,21,25,26,30,38,43,51,57,73,74,75,76,79,80,82,84,91,98,100,102,103,104,105,106,107,111,112,118,119,120,122,125,126,129,132,136,138,139,151,153,162,184,194,195,208,212,222,224,226,234,242,243 |
| Vomiting | 36 | 20,22,25,30,84,98,103,105,112,122,126,132,138,139,141,144,147,151,156,160,162,164,166,175,176,188,190,193,209,217,222,224,227,234,236,242 |
| Nausea | 22 | 22,30,84,98,103,105,112,122,126,132,144,147,156,160,162,164,176,188,190,212,224,242 |
| Diarrhea | 36 | 20,21,22,30,34,43,73,75,79,95,97,98,103,104,109,112,122,124,130,138,144,148,156,160,161,162,165,176,184,193,196,222,224,226,234,236 |
| Anorexia | 17 | 30,34,43,80,98,105,111,122,126,144,153,165,176,196,217,242,243 |
| Cough | 29 | 30,43,84,91,106,115,122,126,132,144,148,153,156,160,161,162,165,176,184,190,193,196,208,212,218,222,224,226,236 |
| Exanthema | 5 | 38,91,96,139,226 |
| Rash | 83 | 12,20,21,22,25,26,30,34,41,43,51,57,63,72,73,74,75,76,78,79,80,82,84,91,95,97,98,99,100,102,103,104,105,106,108,111,112,118,119,120,122,123,124,126,127,129,130,132,138,141,144,146,148,149,151,152,155,156,160,161,162,165,166,175,176,181,184,188,193,194,196,208,209,212,213,217,222,224,226,234,235,236,243 |
| Petechiae | 30 | 21,43,51,60,63,74,90,91,96,100,109,111,118,120,122,123,129,135,139,144,152,155,158,162,176,190,195,209,227,236 |
| Abdominal pain | 61 | 20,22,25,30,34,38,51,54,60,71,72,73,74,75,80,82,84,90,91,95,96,97,98,100,104,105,111,112,113,122,123,124,126,129,130,138,139,141,144,147,148,151,155,156,161,162,164,165,166,175,188,194,196,208,218,226,227,234,235,236,242 |
| Back pain | 9 | 73,74,105,122,132,212,224,236,243 |
| Seizure | 7 | 51,95,97,103,138,151,167 |
| Ascites | 25 | 23,25,41,51,76,90,91,99,108,122,138,144,148,155,162,175,181,193,194,213,217,222,226,227,236 |
| Pleural effusion | 23 | 23,30,41,51,76,78,90,91,99,122,138,148,152,155,162,175,196,213,217,222,226,227,236 |
| Hepatosplenomegaly | 5 | 41,100,115,193,196 |
| Hepatomegaly | 41 | 30,41,51,54,71,74,76,90,95,96,99,108,122,123,124,126,129,130,132,138,144,151,155,162,167,175,176,181,183,190,193,194,209,213,217,222,226,227,234,235,236 |
| Splenomegaly | 20 | 41,76,95,96,99,122,126,132,138,151,152,155,162,175,217,222,226,227,234,236 |
| Shock | 12 | 22,30,41,51,144,152,167,193,207,208,222,227 |
| Icterus/Jaundice | 13 | 51,96,105,126,144,151,175,181,184,190,193,217,222 |
| Lethargy | 5 | 38,71,111,124,194 |
| Epistaxis | 25 | 74,89,91,96,106,122,123,126,127,138,139,141,144,148,151,155,158,162,165,166,209,222,235,236,243 |
| Gingivorrhagia | 16 | 74,97,99,127,138,139,141,144,146,148,151,155,158,162,165,166 |
| Asthenia | 6 | 26,75,119,129,242,243 |
| Lymphadenopathy | 13 | 76,78,122,129,130,132,139,144,152,176,234,235,236 |
| Respiratory disorders | 13 | 21,30,78,91,103,104,108,111,122,130,148,222,226 |
| Myocarditis | 5 | 76,78,134,148,152 |
| Hypotension | 14 | 30,76,78,84,95,96,100,104,130,134,141,155,160,222 |
| Encephalopathy | 8 | 76,78,97,99,108,134,152,190 |
| Itching eruption | 6 | 84,100,109,112,119,176 |
| Pruritus | 5 | 139,162,165,234,243 |
| Sore throat | 12 | 84,106,115,129,130,144,164,184,196,224,226,236 |
| Melena | 13 | 89,91,97,122,123,138,144,148,162,165,166,227,236 |
| Convulsion | 5 | 99,105,209,222,236 |
| Eye pain | 6 | 25,161,212,218,224,236 |
| Hematuria | 16 | 34,91,96,97,99,122,127,139,144,148,158,166,209,222,235,236 |
| Hematemesis | 18 | 91,96,97,100,122,123,127,138,148,158,162,166,175,193,209,227,235,236 |
| Bleeding/Haemorrhagic manifestations | 58 | 20,21,25,30,38,41,43,51,54,56,60,64,71,72,74,76,78,79,82,83,90,91,96,98,104,105,111,112,119,124,125,126,128,130,132,136,138,141,183,184,188,190,193,196,207,208,212,213,215,217,218,219,222,224,226,227,234,242 |

*PID*, publication identification.

**2. Supplementary Data**

**2.1.** **Supplementary File S1. List of publications included in systematic review and meta-analysis**

1 Zhao, H. et al. Epidemiological and Virological Characterizations of the 2014 Dengue Outbreak in Guangzhou, China. PloS one 11, doi:10.1371/journal.pone.0156548 (2016).

2 Xiao, J. P. et al. Characterizing a large outbreak of dengue fever in Guangdong Province, China. Infect Dis Poverty 5, 44, doi:10.1186/s40249-016-0131-z (2016).

3 Wei, H. Y., Shu, P. Y. & Hung, M. N. Characteristics and risk factors for fatality in patients with dengue Hemorrhagic Fever, Taiwan, 2014. American Journal of Tropical Medicine and Hygiene 95, 322-327 (2016).

4 Villabona-Arenas, C. J. et al. Epidemiological dynamics of an urban Dengue 4 outbreak in Sao Paulo, Brazil. PeerJ 4, doi:10.7717/peerj.1892 (2016).

5 Vairo, F. et al. Clinical, virologic, and epidemiologic characteristics of dengue outbreak, Dar es Salaam, Tanzania, 2014. Emerging infectious diseases 22, 895-899 (2016).

6 Tun, M. M. N. et al. Characterization of the 2013 dengue epidemic in Myanmar with dengue virus 1 as the dominant serotype. Infection Genetics and Evolution 43, 31-37, doi:10.1016/j.meegid.2016.04.025 (2016).

7 Tittarelli, E., Barrero, P. R., Mistchenko, A. S. & Valinotto, L. E. Secondary dengue virus infections during the 2009 outbreak in Buenos Aires. Tropical Medicine & International Health 21, 28-32, doi:10.1111/tmi.12619 (2016).

8 Thomas, D. L. et al. Reemergence of dengue in Southern Texas, 2013. Emerging infectious diseases 22, 1002-1007 (2016).

9 Tazeen, A. et al. Occurrence of co-infection with dengue viruses during 2014 in New Delhi, India. Epidemiology and infection, 1-11 (2016).

10 Sun, J. et al. The epidemiological characteristics and genetic diversity of dengue virus during the third largest historical outbreak of dengue in Guangdong, China, in 2014. The Journal of infection 72, 80-90, doi:10.1016/j.jinf.2015.10.007 (2016).

11 Suleman, M. et al. Dengue outbreak in Swat and Mansehra, Pakistan 2013; an epidemiological and diagnostic perspective. Asian Pacific Journal of Tropical Medicine 9, 371-375, doi:10.1016/j.apjtm.2016.03.010 (2016).

12 Succo, T. et al. Autochthonous dengue outbreak in Nimes, South of France, July to September 2015. Eurosurveillance 21, 5-11, doi:10.2807/1560-7917.es.2016.21.21.30240 (2016).

13 Siddiqui, O., Chakravarti, A. & Abhishek, K. S. Dengue: Lessons of an outbreak. Journal of Clinical and Diagnostic Research 10, DC01-DC04 (2016).

14 Saha, K. et al. Changing pattern of dengue virus serotypes circulating during 2008-2012 and reappearance of dengue serotype 3 may cause outbreak in Kolkata, India. Journal of medical virology (2016).

15 Quam, M. B., Sessions, O., Kamaraj, U. S., Rocklov, J. & Wilder-Smith, A. Dissecting Japan's Dengue Outbreak in 2014. The American journal of tropical medicine and hygiene 94, 409-412, doi:10.4269/ajtmh.15-0468 (2016).

16 Phommanivong, V. et al. Co-circulation of the dengue with chikungunya virus during the 2013 outbreak in the southern part of Lao PDR. Tropical Medicine and Health 44 (2016).

17 Petitdemange, C. et al. Longitudinal Analysis of Natural Killer Cells in Dengue Virus-Infected Patients in Comparison to Chikungunya and Chikungunya/Dengue Virus-Infected Patients. PLoS neglected tropical diseases 10, e0004499, doi:10.1371/journal.pntd.0004499 (2016).

18 Pessoa, R. et al. Investigation Into an Outbreak of Dengue-like Illness in Pernambuco, Brazil, Revealed a Cocirculation of Zika, Chikungunya, and Dengue Virus Type 1. Medicine 95, e3201, doi:10.1097/MD.0000000000003201 (2016).

19 Onoja, A. B., Adeniji, J. A. & Olaleye, O. D. High rate of unrecognized dengue virus infection in parts of the rainforest region of Nigeria. Acta tropica 160, 39-43, doi:10.1016/j.actatropica.2016.04.007 (2016).

20 Massangaie, M. et al. Clinical and Epidemiological Characterization of the First Recognized Outbreak of Dengue Virus-Type 2 in Mozambique, 2014. The American journal of tropical medicine and hygiene 94, 413-416, doi:10.4269/ajtmh.15-0543 (2016).

21 Mares-Guia, M. A. M. M. et al. Molecular identification of Q fever in patients with a suspected diagnosis of dengue in Brazil in 2013-2014. American Journal of Tropical Medicine and Hygiene 94, 1090-1094 (2016).

22 Lin, Y. P. et al. Clinical and epidemiological features of the 2014 large-scale dengue outbreak in Guangzhou city, China. BMC infectious diseases 16, 102, doi:10.1186/s12879-016-1379-4 (2016).

23 Khurram, M. et al. Ultrasonographic pattern of plasma leak in dengue haemorrhagic fever. Journal of the Pakistan Medical Association 66, 260-264 (2016).

24 Johnston, D. et al. Notes from the Field: Outbreak of Locally Acquired Cases of Dengue Fever--Hawaii, 2015. MMWR. Morbidity and mortality weekly report 65, 34-35, doi:10.15585/mmwr.mm6502a4 (2016).

25 Jones, J. M. et al. Binational Dengue Outbreak Along the United States-Mexico Border - Yuma County, Arizona, and Sonora, Mexico, 2014. Mmwr-Morbidity and Mortality Weekly Report 65, 495-499 (2016).

26 Huang, L. et al. Epidemiology and characteristics of the dengue outbreak in Guangdong, Southern China, in 2014. European journal of clinical microbiology & infectious diseases : official publication of the European Society of Clinical Microbiology 35, 269-277, doi:10.1007/s10096-015-2540-5 (2016).

27 Haryanto, S. et al. The molecular and clinical features of dengue during outbreak in Jambi, Indonesia in 2015 (vol 110, 119, 2016). Pathogens and Global Health 110, doi:10.1080/20477724.2016.1207306 (2016).

28 Hapuarachchi, H. C. et al. Epidemic resurgence of dengue fever in Singapore in 2013-2014: A virological and entomological perspective. BMC infectious diseases 16, doi:10.1186/s12879-016-1606-z (2016).

29 dos Santos Carmo, A. M., Suzuki, R. B., Riquena, M. M., Eterovic, A. & Sperança, M. A. Maintenance of demographic and hematological profiles in a long-lasting dengue fever outbreak: Implications for management. Infectious Diseases of Poverty 5 (2016).

30 Dhanoa, A. et al. Impact of dengue virus (DENV) co-infection on clinical manifestations, disease severity and laboratory parameters. BMC infectious diseases 16, doi:10.1186/s12879-016-1731-8 (2016).

31 de Teive e Argolo, A. F. L. et al. High frequency of pre-existing neutralizing antibody responses in patients with dengue during an outbreak in Central Brazil. BMC infectious diseases 16 (2016).

32 Cunha, M. D. et al. Phylodynamics of DENV-1 reveals the spatiotemporal co-circulation of two distinct lineages in 2013 and multiple introductions of dengue virus in Goias, Brazil. Infection Genetics and Evolution 43, 130-134, doi:10.1016/j.meegid.2016.05.021 (2016).

33 Chetry, S., Khan, S. A., Apum, B. & Dutta, P. Circulation of dengue virus-1 Genotype III during 2015 dengue outbreak in Arunachal Pradesh: A maiden report from Northeast India. International Journal of Infectious Diseases 45, 433-434, doi:10.1016/j.ijid.2016.02.921 (2016).

34 Chen, C. M. et al. The outcomes of patients with severe dengue admitted to intensive care units. Medicine 95, doi:10.1097/md.0000000000004376 (2016).

35 Chen, B., Yang, J., Luo, L., Yang, Z. & Liu, Q. Who is vulnerable to dengue fever? A community survey of the 2014 outbreak in Guangzhou, China. International journal of environmental research and public health 13 (2016).

36 Chang, S. F. et al. Laboratory-Based Surveillance and Molecular Characterization of Dengue Viruses in Taiwan, 2014. American Journal of Tropical Medicine and Hygiene 94, 804-811, doi:10.4269/ajtmh.15-0534 (2016).

37 Castellanos, J. E. et al. Description of high rates of unapparent and simultaneous multiple dengue virus infection in a Colombian jungle settlement. Tropical biomedicine 33, 375-382 (2016).

38 Buonora, S. N. et al. Accuracy of clinical criteria and an immunochromatographic strip test for dengue diagnosis in a DENV-4 epidemic. BMC infectious diseases 16, 37, doi:10.1186/s12879-016-1368-7 (2016).

39 Andrade, E. H. P. et al. Spatial-temporal co-circulation of dengue virus 1, 2, 3, and 4 associated with coinfection cases in a hyperendemic area of Brazil: A 4-week survey. American Journal of Tropical Medicine and Hygiene 94, 1080-1084 (2016).

40 Ali, A., Ahmad, H., Idrees, M., Zahir, F. & Ali, I. Circulating serotypes of dengue virus and their incursion into non-endemic areas of Pakistan; a serious threat. Virology journal 13, doi:10.1186/s12985-016-0603-6 (2016).

41 Acharyya, A. et al. The dengue fever and its complication: A scenario in a tertiary-level hospital of greater Kolkata. Annals of Tropical Medicine and Public Health 9, 92-96 (2016).

42 Wang, B. et al. The distinct distribution and phylogenetic characteristics of dengue virus serotypes/genotypes during the 2013 outbreak in Yunnan, China. Phylogenetic characteristics of 2013 dengue outbreak in Yunnan, China. Infection, Genetics and Evolution 37, 1-7 (2016).

43 Wang, W. et al. Reemergence and Autochthonous Transmission of Dengue Virus, Eastern China, 2014. Emerging infectious diseases 21, 1670-1673, doi:10.3201/eid2109.150622 (2015).

44 Wang, T. et al. Evaluation of Inapparent Dengue Infections During an Outbreak in Southern China. PLoS neglected tropical diseases 9, doi:10.1371/journal.pntd.0003677 (2015).

45 Wang, S.-F. et al. Large Dengue virus type 1 outbreak in Taiwan. Emerging Microbes & Infections 4, doi:10.1038/emi.2015.46 (2015).

46 Toan, D. T., Hoat, L. N., Hu, W., Wright, P. & Martens, P. Risk factors associated with an outbreak of dengue fever/dengue haemorrhagic fever in Hanoi, Vietnam. Epidemiology and infection 143, 1594-1598, doi:10.1017/s0950268814002647 (2015).

47 Le Viet, T. et al. A dengue outbreak on a floating village at Cat Ba Island in Vietnam. BMC public health 15, 940, doi:10.1186/s12889-015-2235-y (2015).

48 Takamatsu, Y. et al. A Dengue virus serotype 4-dominated outbreak in central Vietnam, 2013. Journal of Clinical Virology 66, 24-26 (2015).

49 Saswat, T. et al. High rates of co-infection of Dengue and Chikungunya virus in Odisha and Maharashtra, India during 2013. Infection, genetics and evolution : journal of molecular epidemiology and evolutionary genetics in infectious diseases 35, 134-141, doi:10.1016/j.meegid.2015.08.006 (2015).

50 Sasaki, T. et al. Susceptibility of Indigenous and Transplanted Mosquito Spp. to Dengue Virus in Japan. Japanese journal of infectious diseases 68, 425-427, doi:10.7883/yoken.JJID.2014.511 (2015).

51 Sahana, K. S. & Sujatha, R. Clinical Profile of Dengue Among Children According to Revised WHO Classification: Analysis of a 2012 Outbreak from Southern India. Indian Journal of Pediatrics 82, 109-113, doi:10.1007/s12098-014-1523-3 (2015).

52 Kim Lien, P. T. et al. Role of Aedes aegypti and Aedes albopictus during the 2011 dengue fever epidemics in Hanoi, Vietnam. Asian Pacific Journal of Tropical Medicine 8, 543-548 (2015).

53 Pech Torres, R. E., Cedillo Rivera, R. M., Loroño Pino, M. A. & Sánchez Burgos, G. G. Serum levels of IFN-β are associated with days of evolution but not with severity of dengue. Journal of medical virology (2015).

54 Palanivel, H., Nair, S., Subramaniyan, A., Ratnam, P. V. & Kanungo, R. Dengue virus infection: Need for appropriate laboratory tests for diagnosis and management of the condition in children during an outbreak. Indian journal of pathology & microbiology 58, 328-331, doi:10.4103/0377-4929.162865 (2015).

55 Ng, L. C. et al. 2013 Dengue Outbreaks in Singapore and Malaysia Caused by Different Viral Strains. American Journal of Tropical Medicine and Hygiene 92, 1150-1155, doi:10.4269/ajtmh.14-0588 (2015).

56 Leduc-Galindo, D. et al. Characterization of the dengue outbreak in Nuevo Leon state, Mexico, 2010 (vol 43, pg 201, 2015). Infection 43, 259-259, doi:10.1007/s15010-015-0739-0 (2015).

57 Kunwar, C. R. & Prakash, R. Dengue outbreak in a large military station: Have we learnt any lesson? Medical Journal Armed Forces India 71, 11-14 (2015).

58 Guo, X. et al. Molecular Characterization and Viral Origin of the First Dengue Outbreak in Xishuangbanna, Yunnan Province, China, 2013. The American journal of tropical medicine and hygiene 93, 390-393, doi:10.4269/ajtmh.14-0044 (2015).

59 Furuya, H. Estimation of reproduction number and probable vector density of the first autochthonous dengue outbreak in Japan in the last 70 years. Environmental Health and Preventive Medicine 20, 466-471 (2015).

60 Ellis, E. M. et al. A household serosurvey to estimate the magnitude of a dengue outbreak in Mombasa, Kenya, 2013. PLoS neglected tropical diseases 9, e0003733, doi:10.1371/journal.pntd.0003733 (2015).

61 de Matos, A. M. et al. CD8+ T Lymphocyte Expansion, Proliferation and Activation in Dengue Fever. PLoS neglected tropical diseases 9 (2015).

62 da Silva Pessoa Vieira, C. J. et al. Detection of Mayaro virus infections during a dengue outbreak in Mato Grosso, Brazil. Acta tropica 147, 12-16 (2015).

63 Barde, P. V. et al. Emergence of dengue in tribal villages of mandla district, Madhya Pradesh, India. Indian Journal of Medical Research, Supplement 141, 584-590 (2015).

64 Zhang, F. C. et al. Severe dengue outbreak in Yunnan, China, 2013. International Journal of Infectious Diseases 27, e4-e6 (2014).

65 Williams, M. et al. Lineage II of Southeast Asian/American DENV-2 is associated with a severe dengue outbreak in the Peruvian Amazon. The American journal of tropical medicine and hygiene 91, 611-620, doi:10.4269/ajtmh.13-0600 (2014).

66 Wilder-Smith, A. et al. The 2012 dengue outbreak in Madeira: Exploring the origins. Eurosurveillance 19 (2014).

67 Vazquez, S. et al. Dengue specific immunoglobulins M, A, and E in primary and secondary dengue 4 infected Salvadorian children. Journal of medical virology 86, 1576-1583, doi:10.1002/jmv.23833 (2014).

68 Tsegaye, M. M. Virological and serological investigation of the first dengue fever outbreak in Ethiopia. American Journal of Tropical Medicine and Hygiene 91, 53 (2014).

69 Thomas, D. L. Re-emergence of dengue in South Texas, 2013. American Journal of Tropical Medicine and Hygiene 91, 387 (2014).

70 Stewart-Ibarra, A. M. et al. Spatiotemporal clustering, climate periodicity, and social-ecological risk factors for dengue during an outbreak in Machala, Ecuador, in 2010. BMC infectious diseases 14, 610, doi:10.1186/s12879-014-0610-4 (2014).

71 Sharp, T. M. et al. Characteristics of a dengue outbreak in a remote pacific island chain--Republic of The Marshall Islands, 2011-2012. PloS one 9, e108445, doi:10.1371/journal.pone.0108445 (2014).

72 Saqib, M. A., Rafique, I., Bashir, S. & Salam, A. A. A retrospective analysis of dengue fever case management and frequency of co-morbidities associated with deaths. BMC research notes 7, 205, doi:10.1186/1756-0500-7-205 (2014).

73 Rezza, G. et al. Co-circulation of dengue and chikungunya viruses, Al Hudaydah, Yemen, 2012. Emerging infectious diseases 20, 1351-1354 (2014).

74 Pozo-Aguilar, J. O. et al. Evaluation of host and viral factors associated with severe dengue based on the 2009 WHO classification. Parasites and Vectors 7 (2014).

75 Parreira, R. et al. Angola’s 2013 dengue outbreak: Clinical, laboratory and molecular analyses of cases from four Portuguese institutions. Journal of Infection in Developing Countries 8, 1210-1215 (2014).

76 Pal, P., Giri, P. P. & Ramanan, A. V. Dengue associated hemophagocytic lymphohistiocytosis: A case series. Indian pediatrics 51, 496-497 (2014).

77 Ocwieja, K. E. et al. Phylogeography and molecular epidemiology of an epidemic strain of dengue virus type 1 in Sri Lanka. The American journal of tropical medicine and hygiene 91, 225-234, doi:10.4269/ajtmh.13-0523 (2014).

78 Neeraja, M. et al. Unusual and rare manifestations of dengue during a dengue outbreak in a tertiary care hospital in South India. Arch Virol 159, 1567-1573, doi:10.1007/s00705-014-2010-x (2014).

79 Naskar, A. et al. A profile of dengue outbreak in adults of an eastern state of India. International Journal of Infectious Diseases 21, 442 (2014).

80 Murad, H., Asahar, R. J., Zaheen, M. & Shawali, R. Outbreak investigation of Dengue fever in Sundia, Chakaiser, Shangla, Pakistan-2008. Journal of Ayub Medical College, Abbottabad : JAMC 26, 571-576 (2014).

81 Mogeni, D. O. et al. Use of integrated disease surveillance and response system (IDSR) to determine the extent of dengue fever outbreak along the coastal region of Kenya. American Journal of Tropical Medicine and Hygiene 91, 247-248 (2014).

82 Martins Vdo, C. et al. Clinical and virological descriptive study in the 2011 outbreak of dengue in the Amazonas, Brazil. PloS one 9, e100535, doi:10.1371/journal.pone.0100535 (2014).

83 Kyobe Bosa, H., Montgomery, J. M., Kimuli, I. & Lutwama, J. J. Dengue fever outbreak in Mogadishu, Somalia 2011: Co-circulation of three dengue virus serotypes. International Journal of Infectious Diseases 21, 3 (2014).

84 Khan, S. A., Dutta, P., Topno, R., Soni, M. & Mahanta, J. Dengue outbreak in a hilly state of Arunachal Pradesh in Northeast India. The Scientific World Journal 2014 (2014).

85 Jindal, N., Bansal, R. & Dhuria, N. The 2011 outbreak of dengue virus infection in Malwa region of Punjab, India-an evaluation of various diagnostic tests. Asian Pacific Journal of Tropical Disease 4, 363-366 (2014).

86 Hunsperger, E. et al. Dengue virus infection among members of the Uganda people's defense force. American Journal of Tropical Medicine and Hygiene 91, 430-431 (2014).

87 Huang, X. Y. et al. Outbreak of dengue Fever in central China, 2013. Biomedical and environmental sciences : BES 27, 894-897, doi:10.3967/bes2014.125 (2014).

88 Barcelos Figueiredo, L. et al. Dengue virus 2 American-Asian genotype identified during the 2006/2007 outbreak in Piaui, Brazil reveals a Caribbean route of introduction and dissemination of dengue virus in Brazil. PloS one 9, e104516, doi:10.1371/journal.pone.0104516 (2014).

89 Faye, O. et al. Urban epidemic of dengue virus serotype 3 infection, Senegal, 2009. Emerging infectious diseases 20, 456-459, doi:10.3201/eid2003.121885 (2014).

90 Fariz-Safhan, M. N., Tee, H. P., Abu Dzarr, G. A., Sapari, S. & Lee, Y. Y. Bleeding outcome during a dengue outbreak in 2005 in the East-coast region of Peninsular Malaysia: a prospective study. Tropical biomedicine 31, 270-280 (2014).

91 Cavalcanti, L. P. Evaluation of the WHO classification of dengue disease severity during an epidemic in 2011 in the state of Ceará, Brazil. Memorias do Instituto Oswaldo Cruz 109, 93-98 (2014).

92 Carvalho, R. M. & Nascimento, L. F. Space-time description of dengue outbreaks in Cruzeiro, Sao Paulo, in 2006 and 2011. Revista da Associacao Medica Brasileira (1992) 60, 565-570, doi:10.1590/1806-9282.60.06.016 (2014).

93 Deligny, C. et al. Dengue fever in patients under biologics. Journal of Clinical Virology 61, 442-443 (2014).

94 Chen, R. F. et al. Augmented miR-150 expression associated with depressed SOCS1 expression involved in dengue haemorrhagic fever. Journal of Infection 69, 366-374 (2014).

95 Chatterjee, N. et al. An observational study of dengue fever in a tertiary care hospital of eastern India. Journal of Association of Physicians of India 62, 224-227 (2014).

96 Assir, M. Z. K., Masood, M. A. & Ahmad, H. I. Concurrent dengue and malaria infection in Lahore, Pakistan during the 2012 dengue outbreak. International Journal of Infectious Diseases 18, 41-46 (2014).

97 Assir, M. Z., Ahmad, H. I., Masood, M. A., Kamran, U. & Yusuf, N. W. Deaths due to dengue fever at a tertiary care hospital in Lahore, Pakistan. Scandinavian journal of infectious diseases 46, 303-309, doi:10.3109/00365548.2013.877155 (2014).

98 Allonso, D., Meneses, M. D. F., Fernandes, C. A., Ferreira, D. F. & Mohana-Borges, R. Assessing positivity and circulating levels of NS1 in samples from a 2012 dengue outbreak in Rio de Janeiro, Brazil. PloS one 9 (2014).

99 VinodKumar, C. S. et al. Episode of coexisting infections with multiple dengue virus serotypes in central Karnataka, India. Journal of infection and public health 6, 302-306 (2013).

100 Thangaratham, P. S. et al. Clinical spectrum during dengue haemorrhagic fever epidemics in Tirupur (India). Journal of vector borne diseases 50, 311-313 (2013).

101 Taulung, L. A. et al. Dengue outbreak - Federated States of Micronesia, 2012-2013. Morbidity and Mortality Weekly Report 62, 570-573 (2013).

102 Schwartz, E. et al. Detection on four continents of dengue fever cases related to an ongoing outbreak in Luanda, Angola, March to May 2013. Eurosurveillance 18, 20488 (2013).

103 Pun, S. B. & Shah, Y. Critical phase among patients with dengue fever during the 2010 outbreak in Nepal. Transactions of the Royal Society of Tropical Medicine and Hygiene 107, 598-600, doi:10.1093/trstmh/trt061 (2013).

104 Neeraja, M., Lakshmi, V., Dash, P. K., Parida, M. M. & Rao, P. V. L. The clinical, serological and molecular diagnosis of emerging dengue infection at a tertiary care institute in Southern, India. Journal of Clinical and Diagnostic Research 7, 457-461 (2013).

105 Madani, T. A. et al. Outbreak of viral hemorrhagic fever caused by dengue virus type 3 in Al-Mukalla, Yemen. BMC infectious diseases 13 (2013).

106 Lorenzi, O. D. et al. Acute febrile illness surveillance in a tertiary hospital emergency department: comparison of influenza and dengue virus infections. The American journal of tropical medicine and hygiene 88, 472-480, doi:10.4269/ajtmh.12-0373 (2013).

107 Khan, S. A. et al. Dengue outbreak in an Indo-Myanmar boarder area: Epidemiological aspects and risk factors. Tropical biomedicine 30, 451-458 (2013).

108 Kalappanvar, N. K., VinodKumar, C. S., Basavarajappa, K. G., Chandrasekhar, G. & Sanjay, D. Outbreak of dengue infection in rural Davangere, Karnataka. Asian Pac J Trop Med 6, 502-503, doi:10.1016/s1995-7645(13)60084-x (2013).

109 Ho, T. S., Wang, S. M., Lin, Y. S. & Liu, C. C. Clinical and laboratory predictive markers for acute dengue infection. Journal of Biomedical Science 20 (2013).

110 Goyal, V. et al. Clinical spectrums of dengue fever in a tertiary care centre with particular references to atypical presentation in the 2011 outbreak at Bathinda, Punjab, India. International Journal of Pharmacy and Pharmaceutical Sciences 5, 363-367 (2013).

111 Fahri, S. et al. Molecular Surveillance of Dengue in Semarang, Indonesia Revealed the Circulation of an Old Genotype of Dengue Virus Serotype-1. PLoS neglected tropical diseases 7, doi:10.1371/journal.pntd.0002354 (2013).

112 Dhar, M. et al. De novo experience of a single outbreak of dengue infection at a tertiary referral centre of Uttarakhand, North India. Journal, Indian Academy of Clinical Medicine 14, 225-229 (2013).

113 Ahmed, S. et al. The 2011 dengue haemorrhagic fever outbreak in Lahore - an account of clinical parameters and pattern of haemorrhagic complications. Journal of the College of Physicians and Surgeons--Pakistan : JCPSP 23, 463-467, doi:07.2013/jcpsp.463467 (2013).

114 Dengue outbreak--Federated States of Micronesia, 2012-2013. MMWR. Morbidity and mortality weekly report 62, 570-573 (2013).

115 Ujwala, U., Naik, J., Rajderkar, S. & Langare, S. Epidemiological investigation of a fever outbreak in girls' hostel, governmental medical college. Journal of Research in Medical Sciences 17, 114-115 (2012).

116 Sun, J. et al. Inapparent infection during an outbreak of dengue fever in southeastern China. Viral Immunology 25, 456-460 (2012).

117 Sousa, C. A. et al. Ongoing outbreak of dengue type 1 in the Autonomous Region of Madeira, Portugal: preliminary report. Euro surveillance : bulletin Europeen sur les maladies transmissibles = European communicable disease bulletin 17 (2012).

118 Peng, H. J. et al. A local outbreak of dengue caused by an imported case in Dongguan China. BMC public health 12, 83 (2012).

119 Nkoghe, D. et al. No clinical or biological difference between Chikungunya and Dengue fever during the 2010 Gabonese outbreak. Infectious Disease Reports 4, 11-13 (2012).

120 Jing, Q. L. et al. Emergence of dengue virus 4 genotype II in Guangzhou, China, 2010: Survey and molecular epidemiology of one community outbreak. BMC infectious diseases 12 (2012).

121 Vedpathak, V. L., Soundale, S. G., Lakde, R. N., Deo, D. S. & Khadilkar, H. A. Clinico-epidemiological profile of an outbreak of dengue fever in rural area of Ambajogai Mandai, district Beed. Journal of Communicable Diseases 43, 69-72 (2011).

122 Mohammad, H., Sarkar, D. N., Amin, M. R., Basher, A. & Ahmed, T. Clinical profile and outcome of patients with dengue syndrome in hospital care. Journal of Medicine 12, 131-138 (2011).

123 Malik, A. et al. Dengue hemorrhagic fever outbreak in children in Port Sudan. Journal of infection and public health 4, 1-6, doi:10.1016/j.jiph.2010.08.001 (2011).

124 Giraldo, D. et al. Characteristics of children hospitalized with dengue fever in an outbreak in Rio de Janeiro, Brazil. Transactions of the Royal Society of Tropical Medicine and Hygiene 105, 601-603, doi:10.1016/j.trstmh.2011.07.007 (2011).

125 Mohammed, H. et al. An outbreak of dengue fever in St. Croix (US Virgin Islands), 2005. PloS one 5, e13729, doi:10.1371/journal.pone.0013729 (2010).

126 Kulkarni, M. J., Sarathi, V., Bhalla, V., Shivpuri, D. & Acharya, U. Clinico-epidemiological profile of children hospitalized with dengue. Indian J Pediatr 77, 1103-1107, doi:10.1007/s12098-010-0202-2 (2010).

127 Humayoun, M. A., Waseem, T., Jawa, A. A., Hashmi, M. S. & Akram, J. Multiple dengue serotypes and high frequency of dengue hemorrhagic fever at two tertiary care hospitals in Lahore during the 2008 dengue virus outbreak in Punjab, Pakistan. International Journal of Infectious Diseases 14, e54-e59 (2010).

128 Tomashek, K. M. et al. Description of a large island-wide outbreak of dengue in Puerto Rico, 2007. American Journal of Tropical Medicine and Hygiene 81, 467-474 (2009).

129 Suharti, C. et al. Hanta virus infection during dengue virus infection outbreak in Indonesia. Acta Med Indones 41, 75-80 (2009).

130 Riaz, M. M. et al. Outbreak of dengue fever in Karachi 2006: a clinical perspective. JPMA. The Journal of the Pakistan Medical Association 59, 339-344 (2009).

131 Meynard, J. B. et al. First description of a dengue fever outbreak in the interior of French Guiana, February 2006. European journal of public health 19, 183-188 (2009).

132 Kularatne, S. A. M., Gihan, M. C., Weerasinghe, S. C. & Gunasena, S. Concurrent outbreaks of Chikungunya and Dengue fever in Kandy, Sri Lanka, 2006-07: a comparative analysis of clinical and laboratory features. Postgraduate Medical Journal 85, 342-346, doi:10.1136/pgmj.2007.066746 (2009).

133 de Araujo, J. M. et al. Quantification of dengue virus type 3 RNA in fatal and non-fatal cases in Brazil, 2002. Transactions of the Royal Society of Tropical Medicine and Hygiene 103, 952-954, doi:10.1016/j.trstmh.2009.01.025 (2009).

134 Thomas, L. et al. Influence of the dengue serotype, previous dengue infection, and plasma viral load on clinical presentation and outcome during a dengue-2 and dengue-4 co-epidemic. The American journal of tropical medicine and hygiene 78, 990-998 (2008).

135 Tang, Y. et al. Unique impacts of HBV co-infection on clinical and laboratory findings in a recent dengue outbreak in China. The American journal of tropical medicine and hygiene 79, 154-158 (2008).

136 Tang, J. W. et al. A wide spectrum of dengue IgM and PCR positivity post-onset of illness found in a large dengue 3 outbreak in Pakistan. Journal of medical virology 80, 2113-2121 (2008).

137 Ratsitorahina, M. et al. Outbreak of dengue and Chikungunya fevers, Toamasina, Madagascar, 2006. Emerging infectious diseases 14, 1135-1137, doi:10.3201/eid1407.071521 (2008).

138 Rai, S. et al. Clinico-laboratory findings of patients during dengue outbreak from a tertiary care hospital in Delhi. Trop Doct 38, 175-177, doi:10.1258/td.2007.070229 (2008).

139 Passos, S. R. L. et al. Clinical and laboratory signs as dengue markers during an outbreak in Rio de Janeiro. Infection 36, 570-574 (2008).

140 Liu, C. C. et al. High case-fatality rate of adults with dengue hemorrhagic fever during an outbreak in non-endemic Taiwan: Risk factors for dengue-infected elders. American Journal of Infectious Diseases 4, 10-17 (2008).

141 Lee, V. J. et al. Predictive value of simple clinical and laboratory variables for dengue hemorrhagic fever in adults. Journal of Clinical Virology 42, 34-39 (2008).

142 Kuo, M. C. et al. Impact of renal failure on the outcome of dengue viral infection. Clinical Journal of the American Society of Nephrology 3, 1350-1356 (2008).

143 Koh, B. K. W. et al. The 2005 dengue epidemic in Singapore: Epidemiology, prevention and control. Annals of the Academy of Medicine Singapore 37, 538-545 (2008).

144 Khan, N. A. et al. Clinical profile and outcome of hospitalized patients during first outbreak of dengue in Makkah, Saudi Arabia. Acta tropica 105, 39-44, doi:10.1016/j.actatropica.2007.09.005 (2008).

145 Khan, E. et al. Co-circulations of two genotypes of dengue virus in 2006 out-break of dengue hemorrhagic fever in Karachi, Pakistan. Journal of clinical virology : the official publication of the Pan American Society for Clinical Virology 43, 176-179, doi:10.1016/j.jcv.2008.06.003 (2008).

146 Ghani, M. H. et al. Dengue virus outbreak in the year 2006 at a tertiary care centre in Sindh. Journal of the Liaquat University of Medical and Health Sciences 7, 71-74 (2008).

147 Dutta, S. et al. Increased utilization of treatment centre facilities during a dengue fever outbreak in Kolkata, India. Dengue Bulletin 32, 162-166 (2008).

148 Chandralekha, Pratyush, G. & Anjan, T. The north Indian dengue outbreak 2006: a retrospective analysis of intensive care unit admissions in a tertiary care hospital. Transactions of the Royal Society of Tropical Medicine and Hygiene 102, 143-147, doi:10.1016/j.trstmh.2007.11.002 (2008).

149 Bharaj, P. et al. Concurrent infections by all four dengue virus serotypes during an outbreak of dengue in 2006 in Delhi, India. Virology journal 5 (2008).

150 Baruah, K., Singh, K. I., Agrawal, C. M. & Dhillon, G. P. S. An outbreak of dengue in Moreh: A small rural town in Manipur near Indo-Myanmar border. Dengue Bulletin 32, 219-221 (2008).

151 Ahmed, S. et al. Dengue fever outbreak in Karachi 2006 - A study of profile and outcome of children under 15 years of age. Journal of the Pakistan Medical Association 58, 4-8 (2008).

152 Zhang, F. et al. A clinical, epidemiological and virological study of a dengue fever outbreak in Guangzhou, China - 2002-2006. Dengue Bulletin 31, 10-18 (2007).

153 Xu, G. et al. An outbreak of dengue virus serotype 1 infection in Cixi, Ningbo, People's Republic of China, 2004, associated with a traveler from Thailand and high density of Aedes albopictus. The American journal of tropical medicine and hygiene 76, 1182-1188 (2007).

154 Wang, L. et al. Implications of dynamic changes among tumor necrosis factor-alpha (TNF-alpha), membrane TNF receptor, and soluble TNF receptor levels in regard to the severity of dengue infection. The American journal of tropical medicine and hygiene 77, 297-302 (2007).

155 Thomas, E. A., John, M. & Bhatia, A. Cutaneous manifestations of dengue viral infection in Punjab (north India). International Journal of Dermatology 46, 715-719, doi:10.1111/j.1365-4632.2007.03298.x (2007).

156 Seet, R. C., Quek, A. M. & Lim, E. C. Post-infectious fatigue syndrome in dengue infection. Journal of clinical virology : the official publication of the Pan American Society for Clinical Virology 38, 1-6, doi:10.1016/j.jcv.2006.10.011 (2007).

157 Samuel, P. P., Thenmozhi, V. & Tyagi, B. K. A focal outbreak of dengue fever in a rural area of Tamil Nadu [1]. Indian Journal of Medical Research 125, 179-181 (2007).

158 Mourao, M. P., Lacerda, M. V., Macedo, V. O. & Santos, J. B. Thrombocytopenia in patients with dengue virus infection in the Brazilian Amazon. Platelets 18, 605-612, doi:10.1080/09537100701426604 (2007).

159 Levi, J. E. et al. Evaluation of a commercial real-time PCR kit for detection of dengue virus in samples collected during an outbreak in Goiania, Central Brazil, in 2005. J Clin Microbiol 45, 1893-1897, doi:10.1128/jcm.00065-07 (2007).

160 Kularatne, S. A., Pathirage, M. M., Kumarasiri, P. V., Gunasena, S. & Mahindawanse, S. I. Cardiac complications of a dengue fever outbreak in Sri Lanka, 2005. Transactions of the Royal Society of Tropical Medicine and Hygiene 101, 804-808, doi:10.1016/j.trstmh.2007.02.021 (2007).

161 Khan, E. et al. Dengue outbreak in Karachi, Pakistan, 2006: experience at a tertiary care center. Transactions of the Royal Society of Tropical Medicine and Hygiene 101, 1114-1119, doi:10.1016/j.trstmh.2007.06.016 (2007).

162 Chowell, G. et al. Clinical diagnostic delays and epidemiology of dengue fever during the 2002 outbreak in Colima, Mexico. Dengue Bulletin 31, 26-35 (2007).

163 Wang, W.-K. et al. Slower rates of clearance of viral load and virus-containing immune complexes in patients with dengue hemorrhagic fever. Clinical Infectious Diseases 43, 1023-1030, doi:10.1086/507635 (2006).

164 Suwandono, A. et al. Four dengue virus serotypes found circulating during an outbreak of dengue fever and dengue haemorrhagic fever in Jakarta, Indonesia, during 2004. Transactions of the Royal Society of Tropical Medicine and Hygiene 100, 855-862 (2006).

165 Lee, M. S., Hwang, K. P., Chen, T. C., Lu, P. L. & Chen, T. P. Clinical characteristics of dengue and dengue hemorrhagic fever in a medical center of southern Taiwan during the 2002 epidemic. Journal of microbiology, immunology, and infection = Wei mian yu gan ran za zhi 39, 121-129 (2006).

166 Kapoor, H. K., Bhai, S., John, M. & Xavier, J. Ocular manifestations of dengue fever in an East Indian epidemic. Canadian journal of ophthalmology. Journal canadien d'ophtalmologie 41, 741-746, doi:10.3129/i06-069 (2006).

167 Hoti, S. L. et al. Dengue and dengue haemorrhagic fever outbreak in Pondicherry, South India, during 2003-2004: Emergence of DENV-3. Dengue Bulletin 30, 42-50 (2006).

168 Hayes, J. M. et al. Risk factors for infection during a dengue-1 outbreak in Maui, Hawaii, 2001. Transactions of the Royal Society of Tropical Medicine and Hygiene 100, 559-566, doi:10.1016/j.trstmh.2005.08.013 (2006).

169 Hati, A. K. Studies on dengue and dengue haemorrhagic fever(DHF) in West Bengal State, India. Journal of Communicable Diseases 38, 124-129 (2006).

170 Hanna, J. N. et al. Multiple outbreaks of dengue serotype 2 in north Queensland, 2003/04. Aust N Z J Public Health 30, 220-225 (2006).

171 Dash, P. K. et al. Reemergence of dengue virus type-3 (subtype-III) in India: implications for increased incidence of DHF & DSS. Virology journal 3, 55, doi:10.1186/1743-422x-3-55 (2006).

172 Dar, L., Gupta, E., Narang, P. & Broor, S. Cocirculation of dengue serotypes, Delhi, India, 2003. Emerging infectious diseases 12, 352-353, doi:10.3201/eid1202.050767 (2006).

173 Chua, S. K. et al. Isolation of monoclonal antibodies-escape variant of dengue virus serotype 1. Singapore Med J 47, 940-946 (2006).

174 Cheah, W. L., Chang, M. S. & Wang, Y. C. Spatial, environmental and entomological risk factors analysis on a rural dengue outbreak in Lundu District in Sarawak, Malaysia. Tropical biomedicine 23, 85-96 (2006).

175 Singh, N. P. et al. The 2003 outbreak of Dengue fever in Delhi, India. The Southeast Asian journal of tropical medicine and public health 36, 1174-1178 (2005).

176 Seet, R. C. S., Eng, E. O., Hwee, B. W. & Paton, N. I. An outbreak of primary dengue infection among migrant Chinese workers in Singapore characterized by prominent gastrointestinal symptoms and a high proportion of symptomatic cases. Journal of Clinical Virology 33, 336-340 (2005).

177 Rodriguez-Roche, R. et al. Dengue virus type 3, Cuba, 2000-2002. Emerging infectious diseases 11, 773-774, doi:10.3201/eid1105.040916 (2005).

178 Rodriguez-Roche, R. et al. Dengue virus type 2 in Cuba, 1997: conservation of E gene sequence in isolates obtained at different times during the epidemic. Arch Virol 150, 415-425, doi:10.1007/s00705-004-0445-1 (2005).

179 Ratho, R. K., Mishra, B., Kaur, J., Kakkar, N. & Sharma, K. An outbreak of dengue fever in periurban slums of Chandigarh, India, with special reference to entomological and climatic factors. Indian journal of medical sciences 59, 518-526 (2005).

180 Peyrefitte, C. N. et al. Dengue type 3 virus, Saint Martin, 2003-2004. Emerging infectious diseases 11, 757-761, doi:10.3201/eid1105.040959 (2005).

181 Itha, S. et al. Profile of liver involvement in dengue virus infection. Natl Med J India 18, 127-130 (2005).

182 Gupta, E., Dar, L., Narang, P., Srivastava, V. K. & Broor, S. Serodiagnosis of dengue during an outbreak at a tertiary care hospital in Delhi. Indian Journal of Medical Research 121, 36-38 (2005).

183 Espinoza-Gómez, F., Díaz-Dueñas, P., Torres-Lepe, C., Cedillo-Nakay, R. A. & Newton-Sánchez, O. A. Clinical pattern of hospitalized patients during a dengue epidemic in Colima, Mexico. Dengue Bulletin 29, 8-17 (2005).

184 Effler, P. V. et al. Dengue fever, Hawaii, 2001-2002. Emerging infectious diseases 11, 742-749, doi:10.3201/eid1105.041063 (2005).

185 Durand, M. A. et al. An outbreak of dengue fever in Yap State. Pacific health dialog : a publication of the Pacific Basin Officers Training Program and the Fiji School of Medicine 12, 99-102 (2005).

186 Dash, P. K., Saxena, P., Abhyankar, A., Bhargava, R. & Jana, A. M. Emergence of dengue virus type-3 in northern India. The Southeast Asian journal of tropical medicine and public health 36, 370-377 (2005).

187 Chakravarti, A. & Kumaria, R. Eco-epidemiological analysis of dengue infection during an outbreak of dengue fever, India. Virology journal 2 (2005).

188 Buchy, P. et al. Secondary dengue virus type 4 infections in Vietnam. Southeast Asian Journal of Tropical Medicine and Public Health 36, 178-185 (2005).

189 Wiwanitkit, V. & Manusvanich, P. Can hematocrit and platelet determination on admission predict shock in hospitalized children with dengue hemorrhagic fever? A clinical observation from a small outbreak. Clinical and applied thrombosis/hemostasis : official journal of the International Academy of Clinical and Applied Thrombosis/Hemostasis 10, 65-67 (2004).

190 Wichmann, O. et al. Risk factors and clinical features associated with severe dengue infection in adults and children during the 2001 epidemic in Chonburi, Thailand. Tropical Medicine and International Health 9, 1022-1029 (2004).

191 Wagatsuma, Y., Breiman, R. F., Hossain, A. & Rahman, M. Dengue fever outbreak in a recreation club, Dhaka, Bangladesh. Emerging infectious diseases 10, 747-750, doi:10.3201/eid1004.030330 (2004).

192 Tran, A. et al. Dengue Spatial and Temporal Patterns, French Guiana, 2001. Emerging infectious diseases 10, 615-621 (2004).

193 Shah, I., Deshpande, G. C. & Tardeja, P. N. Outbreak of dengue in Mumbai and predictive markers for dengue shock syndrome. J Trop Pediatr 50, 301-305 (2004).

194 Pervin, M., Tabassum, S., Ali, M., Mamun, K. Z. & Islam, N. Clinical and laboratory observations associated with the 2000 dengue outbreak in Dhaka, Bangladesh. Dengue Bulletin 28, 96-106 (2004).

195 Lorono-Pino, M. A. et al. Introduction of the American/Asian genotype of dengue 2 virus into the Yucatan State of Mexico. The American journal of tropical medicine and hygiene 71, 485-492 (2004).

196 Lai, P. C. et al. Characteristics of a dengue hemorrhagic fever outbreak in 2001 in Kaohsiung. Journal of microbiology, immunology, and infection = Wei mian yu gan ran za zhi 37, 266-270 (2004).

197 Kumar, M. et al. Unusual emergence of Guate98-like molecular subtype of DEN-3 during 2003 dengue outbreak in Delhi. Dengue Bulletin 28, 161-167 (2004).

198 Chao, D. Y. et al. 1998 dengue hemorrhagic fever epidemic in Taiwan. Emerging infectious diseases 10, 552-554, doi:10.3201/eid1003.020518 (2004).

199 Chadee, D. D., Williams, F. L. & Kitron, U. D. Epidemiology of dengue fever in Trinidad, West Indies: the outbreak of 1998. Annals of tropical medicine and parasitology 98, 305-312, doi:10.1179/000349804225003307 (2004).

200 Wang, W. K., Chao, D. Y., Lin, S. R., King, C. C. & Chang, S. C. Concurrent infections by two dengue virus serotypes among dengue patients in Taiwan. Journal of Microbiology, Immunology and Infection 36, 89-95 (2003).

201 Wang, W. K. et al. High levels of plasma dengue viral load during defervescence in patients with dengue hemorrhagic fever: Implications for pathogenesis. Virology 305, 330-338 (2003).

202 Tuntaprasart, W. et al. Seroepidemiological survey among schoolchildren during the 2000-2001 dengue outbreak of Ratchaburi Province, Thailand. Southeast Asian Journal of Tropical Medicine and Public Health 34, 564-568 (2003).

203 Sukri, N. C. et al. Transmission of epidemic dengue hemorrhagic fever in easternmost Indonesia. American Journal of Tropical Medicine and Hygiene 68, 529-535 (2003).

204 Reiter, P. et al. Texas lifestyle limits transmission of dengue virus. Emerging infectious diseases 9, 86-89 (2003).

205 Perret, C. et al. Dengue-1 virus isolation during first dengue fever outbreak on Easter Island, Chile. Emerging infectious diseases 9, 1465-1467, doi:10.3201/eid0911.020788 (2003).

206 Patumanond, J., Tawichasri, C. & Nopparat, S. Dengue hemorrhagic fever, Uttaradit, Thailand. Emerging infectious diseases 9, 1348-1350, doi:10.3201/eid0910.020681 (2003).

207 Narayanan, M., Aravind, M. A., Ambikapathy, P., Prema, R. & Jeyapaul, M. P. Dengue fever - Clinical and laboratory parameters associated with complications. Dengue Bulletin 27, 108-115 (2003).

208 Liu, J. W. et al. Dengue haemorrhagic fever in Taiwan. Dengue Bulletin 27, 19-24 (2003).

209 Kabilan, L. et al. Dengue disease spectrum among infants in the 2001 dengue epidemic in Chennai, Tamil Nadu, India. Journal of Clinical Microbiology 41, 3919-3921 (2003).

210 Hayes, J. M. et al. Risk factors for infection during a severe dengue outbreak in El Salvador in 2000. American Journal of Tropical Medicine and Hygiene 69, 629-633 (2003).

211 Hanna, J. N. et al. Dengue in north Queensland, 2002. Communicable diseases intelligence 27, 384-389 (2003).

212 Ashford, D. A. et al. Outbreak of dengue fever in Palau, Western Pacific: risk factors for infection. The American journal of tropical medicine and hygiene 69, 135-140 (2003).

213 Rahman, M. et al. First outbreak of dengue hemorrhagic fever, Bangladesh. Emerging infectious diseases 8, 738-740, doi:10.3201/eid0807.010398 (2002).

214 Parida, M. M., Dash, P. K., Upadhyay, C., Saxena, P. & Jana, A. M. Serological & virological investigation of an outbreak of dengue fever in Gwalior, India. Indian Journal of Medical Research 116, 248-254 (2002).

215 Hills, S. L., Piispanen, J. P., Humphreys, J. L. & Foley, P. N. A focal, rapidly-controlled outbreak of dengue fever in two suburbs in Townsville, north Queensland, 2001. Communicable diseases intelligence quarterly report 26, 596-600 (2002).

216 Guzman, M. G. et al. Enhanced severity of secondary dengue-2 infections: death rates in 1981 and 1997 Cuban outbreaks. Revista panamericana de salud publica = Pan American journal of public health 11, 223-227 (2002).

217 Aziz, M. M. et al. Predominance of the DEN-3 genotype during the recent dengue outbreak in Bangladesh. The Southeast Asian journal of tropical medicine and public health 33, 42-48 (2002).

218 Yunus, E. B. et al. Dengue outbreak 2000 in Bangladesh: From speculation to reality and exercises. Dengue Bulletin 25, 15-20 (2001).

219 Rigau-Perez, J. G., Ayala-Lopez, A., Vorndam, A. V. & Clark, G. G. Dengue activity in Puerto Rico during an interepidemic period (1995-1997). The American journal of tropical medicine and hygiene 64, 75-83 (2001).

220 Lin, C. F. et al. Generation of IgM anti-platelet autoantibody in dengue patients. Journal of medical virology 63, 143-149 (2001).

221 Heukelbach, J., de Oliveira, F. A., Kerr-Pontes, L. R. & Feldmeier, H. Risk factors associated with an outbreak of dengue fever in a favela in Fortaleza, north-east Brazil. Tropical medicine & international health : TM & IH 6, 635-642 (2001).

222 Ahmed, F. U. et al. Dengue and dengue haemorrhagic fever in children during the 2000 outbreak in Chittagong, Bangladesh. Dengue Bulletin 25, 33-39 (2001).

223 Murgue, B., Roche, C., Chungue, E. & Deparis, X. Prospective study of the duration and magnitude of viraemia in children hospitalised during the 1996-1997 dengue-2 outbreak in French Polynesia. Journal of medical virology 60, 432-438 (2000).

224 Lyerla, R. et al. A dengue outbreak among camp participants in a Caribbean island, 1995. Journal of travel medicine 7, 59-63 (2000).

225 Guzman, M. G. et al. Epidemiologic studies on dengue in Santiago de Cuba, 1997. American journal of epidemiology 152, 793-799, doi:10.1093/aje/152.9.793 (2000).

226 Wali, J. P. et al. Dengue haemorrhagic fever in adults: A prospective study of 110 cases. Tropical Doctor 29, 27-30 (1999).

227 Guzman, M. G. et al. Fatal dengue hemorrhagic fever in Cuba, 1997. International journal of infectious diseases : IJID : official publication of the International Society for Infectious Diseases 3, 130-135 (1999).

228 Dar, L., Broor, S., Sengupta, S., Xess, I. & Seth, P. The first major outbreak of dengue hemorrhagic fever in Delhi, India. Emerging infectious diseases 5, 589-590 (1999).

229 Savage, H. M. et al. Epidemic of dengue-4 virus in Yap State, Federated States of Micronesia, and implication of Aedes hensilli as an epidemic vector. The American journal of tropical medicine and hygiene 58, 519-524 (1998).

230 Paul, R. E., Patel, A. Y., Mirza, S., Fisher-Hoch, S. P. & Luby, S. P. Expansion of epidemic dengue viral infections to Pakistan. International Journal of Infectious Diseases 2, 197-201 (1998).

231 Morrison, A. C., Getis, A., Santiago, M., Rigau-Perez, J. G. & Reiter, P. Exploratory space-time analysis of reported dengue cases during an outbreak in Florida, Puerto Rico, 1991-1992. The American journal of tropical medicine and hygiene 58, 287-298 (1998).

232 Kouri, G. et al. Reemergence of dengue in Cuba: a 1997 epidemic in Santiago de Cuba. Emerging infectious diseases 4, 89-92, doi:10.3201/eid0401.980111 (1998).

233 Hanna, J. N. et al. Two contiguous outbreaks of dengue type 2 in north Queensland. Medical Journal of Australia 168, 221-225 (1998).

234 Deparis, X., Murgue, B., Roche, C., Cassar, O. & Chungue, E. Changing clinical and biological manifestations of dengue during the dengue-2 epidemic in French Polynesia in 1996/97--description and analysis in a prospective study. Tropical medicine & international health : TM & IH 3, 859-865 (1998).

235 Anuradha, S. et al. The 1996 outbreak of dengue hemorrhagic fever in Delhi, India. The Southeast Asian journal of tropical medicine and public health 29, 503-506 (1998).

236 Richards, A. L. et al. The first reported outbreak of dengue hemorrhagic fever in Irian Jaya, Indonesia. The American journal of tropical medicine and hygiene 57, 49-55 (1997).

237 Griffiths, M., Ritchie, S., Terry, D., Norton, R. & Phillips, D. An outbreak of dengue 2 in the Torres Strait. Communicable diseases intelligence 21, 33 (1997).

238 Murray-Smith, S., Weinstein, P. & Skelly, C. Field epidemiology of an outbreak of dengue fever in Charters Towers, Queensland: are insect screens protective? Aust N Z J Public Health 20, 545-547 (1996).

239 Vasconcelos, P. F. et al. A large epidemic of dengue fever with dengue hemorrhagic cases in Ceara State, Brazil, 1994. Rev Inst Med Trop Sao Paulo 37, 253-255 (1995).

240 Rodriguez-Figueroa, L., Rigau-Perez, J. G., Suarez, E. L. & Reiter, P. Risk factors for dengue infection during an outbreak in Yanes, Puerto Rico in 1991. The American journal of tropical medicine and hygiene 52, 496-502 (1995).

241 Nogueira, R. M. et al. Dengue type 2 outbreak in the south of the state of Bahia, Brazil: laboratorial and epidemiological studies. Revista do Instituto de Medicina Tropical de São Paulo 37, 507-510 (1995).

242 Reynes, J. M., Laurent, A., Deubel, V., Telliam, E. & Moreau, J. P. The first epidemic of dengue hemorrhagic fever in French Guiana. The American journal of tropical medicine and hygiene 51, 545-553 (1994).

243 Phillips, I. et al. First documented outbreak of dengue in the Peruvian Amazon region. Bulletin of the Pan American Health Organization 26, 201-207 (1992)

**2.2. Supplementary File S2. PRISMA 2009 Checklist**

| **Section/topic** | **#** | **Checklist item** | **Reported on page #** |
| --- | --- | --- | --- |
| **TITLE** | | |  |
| Title | 1 | Identify the report as a systematic review, meta-analysis, or both. | 1 |
| **ABSTRACT** | | |  |
| Structured summary | 2 | Provide a structured summary including, as applicable: background; objectives; data sources; study eligibility criteria, participants, and interventions; study appraisal and synthesis methods; results; limitations; conclusions and implications of key findings; systematic review registration number. | 2 |
| **INTRODUCTION** | | |  |
| Rationale | 3 | Describe the rationale for the review in the context of what is already known. | 2-3 |
| Objectives | 4 | Provide an explicit statement of questions being addressed with reference to participants, interventions, comparisons, outcomes, and study design (PICOS). | 3 |
| **METHODS** | | |  |
| Protocol and registration | 5 | Indicate if a review protocol exists, if and where it can be accessed (e.g., Web address), and, if available, provide registration information including registration number. | / |
| Eligibility criteria | 6 | Specify study characteristics (e.g., PICOS, length of follow-up) and report characteristics (e.g., years considered, language, publication status) used as criteria for eligibility, giving rationale. | 3-4 |
| Information sources | 7 | Describe all information sources (e.g., databases with dates of coverage, contact with study authors to identify additional studies) in the search and date last searched. | 3 |
| Search | 8 | Present full electronic search strategy for at least one database, including any limits used, such that it could be repeated. | 3 |
| Study selection | 9 | State the process for selecting studies (i.e., screening, eligibility, included in systematic review, and, if applicable, included in the meta-analysis). | 3-4 |
| Data collection process | 10 | Describe method of data extraction from reports (e.g., piloted forms, independently, in duplicate) and any processes for obtaining and confirming data from investigators. | 4 |
| Data items | 11 | List and define all variables for which data were sought (e.g., PICOS, funding sources) and any assumptions and simplifications made. | 4 |
| Risk of bias in individual studies | 12 | Describe methods used for assessing risk of bias of individual studies (including specification of whether this was done at the study or outcome level), and how this information is to be used in any data synthesis. | / |
| Summary measures | 13 | State the principal summary measures (e.g., risk ratio, difference in means). | 4 |
| Synthesis of results | 14 | Describe the methods of handling data and combining results of studies, if done, including measures of consistency (e.g., I^2^) for each meta-analysis. | 4 |
| Risk of bias across studies | 15 | Specify any assessment of risk of bias that may affect the cumulative evidence (e.g., publication bias, selective reporting within studies). | 4 |
| Additional analyses | 16 | Describe methods of additional analyses (e.g., sensitivity or subgroup analyses, meta-regression), if done, indicating which were pre-specified. | 4 |
| **RESULTS** | | |  |
| Study selection | 17 | Give numbers of studies screened, assessed for eligibility, and included in the review, with reasons for exclusions at each stage, ideally with a flow diagram. | 4 |
| Study characteristics | 18 | For each study, present characteristics for which data were extracted (e.g., study size, PICOS, follow-up period) and provide the citations. | 4-5 |
| Risk of bias within studies | 19 | Present data on risk of bias of each study and, if available, any outcome level assessment (see item 12). | / |
| Results of individual studies | 20 | For all outcomes considered (benefits or harms), present, for each study: (a) simple summary data for each intervention group (b) effect estimates and confidence intervals, ideally with a forest plot. | 4-7 |
| Synthesis of results | 21 | Present results of each meta-analysis done, including confidence intervals and measures of consistency. | 4-7 |
| Risk of bias across studies | 22 | Present results of any assessment of risk of bias across studies (see Item 15). | / |
| Additional analysis | 23 | Give results of additional analyses, if done (e.g., sensitivity or subgroup analyses, meta-regression [see Item 16]). | 4-7 |
| **DISCUSSION** | | |  |
| Summary of evidence | 24 | Summarize the main findings including the strength of evidence for each main outcome; consider their relevance to key groups (e.g., healthcare providers, users, and policy makers). | 7-10 |
| Limitations | 25 | Discuss limitations at study and outcome level (e.g., risk of bias), and at review-level (e.g., incomplete retrieval of identified research, reporting bias). | 9-10 |
| Conclusions | 26 | Provide a general interpretation of the results in the context of other evidence, and implications for future research. | 10 |
| **FUNDING** | | |  |
| Funding | 27 | Describe sources of funding for the systematic review and other support (e.g., supply of data); role of funders for the systematic review. | 12 |
